# Artificial intelligence in immunotherapy: revolutionizing diagnostic and therapeutic applications in cancer and autoimmune diseases

**DOI:** 10.1007/s10238-026-02107-5

**Published:** 2026-03-06

**Authors:** Jamal Alshorman, Mohammad Javad Mehran, Yadollah Bahrami, Sara Mohammadzadeh, Rambod Barzigar, Mahdi Morshedi, Khawaja Husnain Haider, Kingsley Miyanda Tembo, Shan-Jie Rong, Nasir Jadgal, Ruba Altahla, Mansoor Bolideei, Yongping Wang

**Affiliations:** 1 Department of Orthopaedics, the Second Affiliated Hospital of Hainan Medical University, Haikou, 570311 China; 2 Department of Biotechnology, JSS Research Foundation, SJCE Technical CampusUniversity of Mysore, Mysore, 570006 Karnataka India; 3 Department of Medical Biotechnology, Faculty of Medicine, Kermanshah University of Medical Sciences, Kermanshah, 6714415185 Iran; 4 Medical Biology Research Center, Kermanshah University of Medical Sciences, Kermanshah, Iran; 5 Advanced Marine Biomanufacturing Laboratory, Centre for Marine Bioproducts Development, College of Medicine and Public Health, Flinders University, Adelaide, SA 5042 Australia; 6 Department of Medical Biotechnology, School of Medicine, College of Medicine and Public Health, Flinders University, Adelaide, SA 5042 Australia; 7 Sulaiman AlRajhi Medical School, Al Bukayriyah, AlQaseem, 52726 Kingdom of Saudi Arabia; 8 Department of Respiratory and Critical Care Medicine, the Center for Biomedical Research, NHC Key Laboratory of Respiratory Diseases, Tongji Hospital, Tongji Medical College, Huazhong University of Sciences and Technology, Wuhan, China; 9 Healit Research International and Trace Research & Innovation, Untold Global Healit Zambia, Lusaka, Zambia; 10 Depaertment of Nursing, Chabahar University of Medical Sciences, Chabahar, Iran; 11 Department of Immunology, Chabahar University of Medical Sciences, Chabahar, Iran

**Keywords:** Artificial Intelligence, Immunotherapy, Cancer, Autoimmune Diseases, Precision Medicine, Machine Learning

## Abstract

Artificial intelligence (AI) is increasingly advancing precision immunotherapy by integrating high-dimensional biomedical data to support diagnosis, treatment selection, and longitudinal monitoring in both cancer and autoimmune diseases. This review summarizes AI applications in biomarker discovery, prediction of immune checkpoint inhibitor (ICI) response and toxicity, neoantigen prioritization, CAR-T cell optimization, and therapeutic antibody engineering. In oncology, multimodal models combining multi-omics, medical imaging, and clinical variables improve patient stratification and non-invasive response assessment, with several imaging- and pathology-based prediction tasks reporting clinically meaningful performance (frequently AUC ~ 0.70–0.95 across tumor types and endpoints). In autoimmune diseases, AI enables earlier diagnosis, molecular subtyping, treatment-response prediction, and real-time disease activity tracking using EHR, laboratory, imaging, and wearable data—supporting precision management in conditions such as rheumatoid arthritis and type 1 diabetes. Key challenges include data heterogeneity, model interpretability, and governance; however, explainable AI, federated learning, and digital twin frameworks offer practical routes toward trustworthy clinical translation. Overall, AI is emerging as a foundational technology for next-generation, patient-specific immunotherapy across oncology and autoimmune medicine.

**Graphical abstract:**

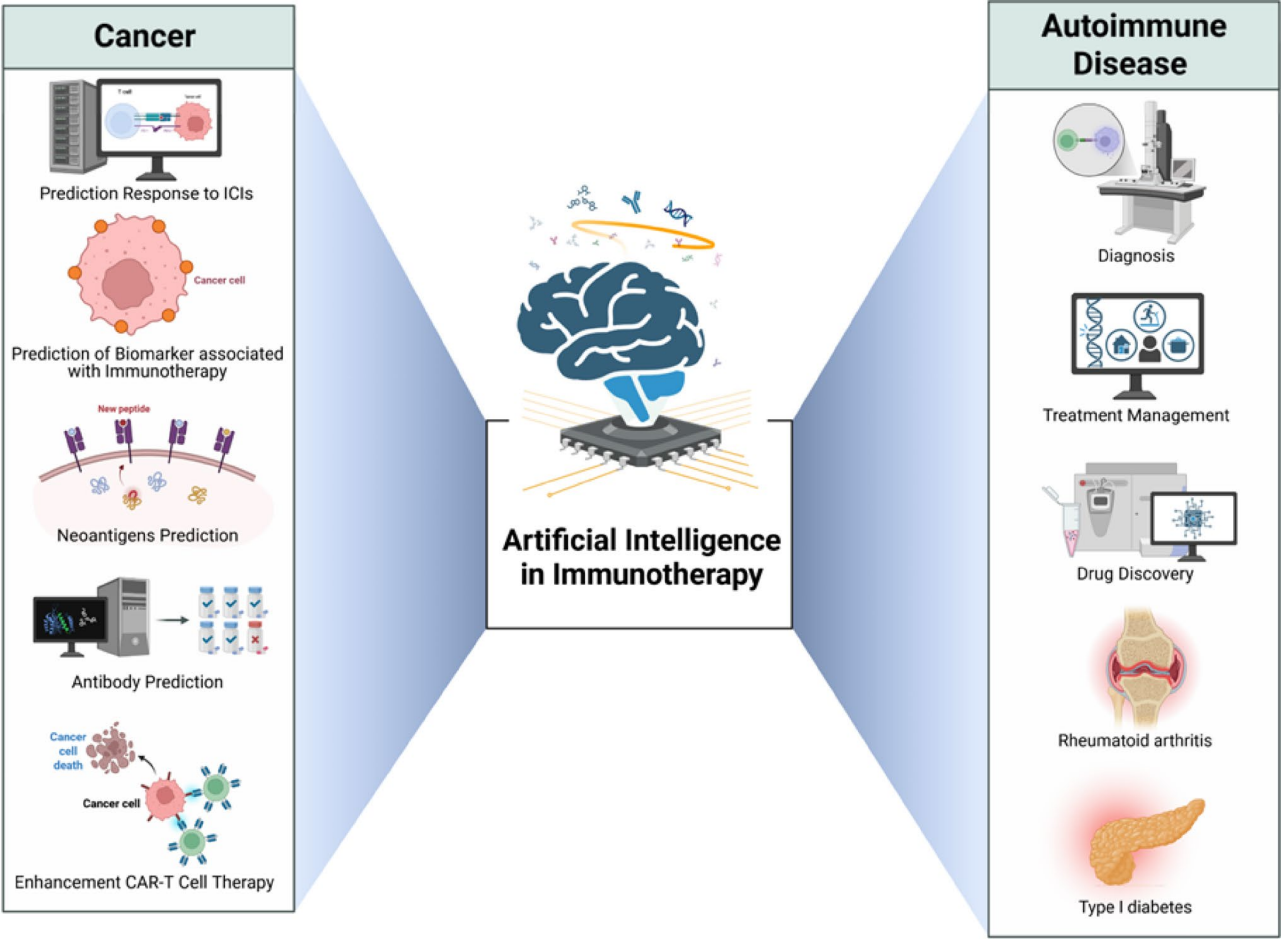

Artificial intelligence enhances cancer and autoimmune disease immunotherapy through biomarker discovery, response prediction, neoantigen and antibody optimization, and real-time treatment management, enabling precision medicine across clinical applications.

## Introduction

Immunotherapy encompasses a wide range of strategies that harness components of the immune system, such as antibodies, immune cells, and cytokines, to treat cancers and autoimmune conditions. It also includes immunomodulation through vaccines aimed at preventing and managing infectious and allergic diseases(1). In this review, “precision immunotherapy” refers to the biomarker-informed selection and dynamic adaptation of immune-based interventions for an individual patient, integrating tumor/immune characteristics, microenvironmental context, and longitudinal monitoring of response and toxicity(2). Accordingly, we cover representative AI approaches ranging from classical machine learning (e.g., regularized regression, random forests, support vector machines) to deep learning architectures—including convolutional neural networks (CNNs) for radiology and whole-slide pathology, and transformer-based models/protein language models for sequence- and structure-informed prediction—applied across multi-omics (including single-cell RNA-seq), immunopeptidomics, medical imaging, digital pathology, and real-world clinical data such as electronic health records (EHRs) and wearable-derived signals.

Cancer remains a major global health concern, causing nearly 10 million deaths each year and ranking as the second leading cause of mortality worldwide. This burden is influenced by aging populations, lifestyle changes, and exposure to risk factors such as tobacco use, obesity, and physical inactivity(3). Conventional cancer treatments, including surgery, chemotherapy, and radiotherapy, remain the foundation of care but face significant limitations. Chemotherapy, although effective against rapidly dividing tumor cells, lacks specificity and harms healthy tissues, leading to adverse effects and a reduced quality of life. Resistance to chemotherapeutic agents, whether intrinsic or acquired, further compromises long-term outcomes. Radiotherapy provides greater precision but can still damage adjacent tissues, causing complications such as skin injury and fatigue. These drawbacks underscore the urgent need for more innovative, targeted, and less toxic therapeutic alternatives(4, 5).

Cancer immunotherapy, which activates the host immune system to recognize and eliminate malignant cells, has emerged as one of the most promising therapeutic strategies.

Tumors frequently evade immune surveillance by exploiting immune checkpoint pathways, which suppress T-cell activation. Clinically relevant checkpoint molecules include programmed death-1 (PD-1), its ligand PD-L1, and cytotoxic T-lymphocyte-associated antigen 4 (CTLA-4)(6, 7). Significant clinical advances in immunotherapy include immune checkpoint inhibitors (ICIs), chimeric antigen receptor (CAR) T cell therapies, therapeutic cancer vaccines, and adoptive cell transfer techniques(8, 9). Conceptually, AI supports immunotherapy along two complementary axes: (i) target discovery and therapeutic engineering (e.g., antigen/neoantigen prioritization, CAR construct optimization, and antibody design), and (ii) clinical decision support (e.g., integrating multimodal patient data to predict ICI response/toxicity, guide treatment selection and sequencing, and enable monitoring and adaptive management)(10).

AI is increasingly integral to advancing cancer immunotherapy by refining target discovery, predicting treatment outcomes, and enabling personalized therapeutic strategies (11). For instance, researchers at the Cleveland Clinic and IBM demonstrated that AI models simulating peptide antigen dynamics can identify viable immunotherapy targets more efficiently than conventional trial-and-error approaches, particularly by capturing antigen structural changes in influencing immune recognition(12). AI-driven platforms now integrate diverse datasets, including genomic, proteomic, and imaging data, to predict patient responses to ICIs and optimize combination strategies(13). These approaches improve diagnostic accuracy by identifying biomarkers, such as PD-L1 expression and tumor mutational burden (TMB), and facilitate the discovery of novel therapeutic targets, like neoantigens(14, 15). Such tools support the design of personalized treatment regimens, enhancing efficacy while reducing adverse effects. Nonetheless, challenges persist in data standardization, model interpretation, and clinical integration, which must be addressed to fully realize AI’s transformative potential in precision oncology(16).

AI also plays a critical role in the diagnosis and management of autoimmune diseases through precise risk assessment and individualized therapeutic planning(17). Autoimmune disorders result from the immune system’s failure to distinguish between self and non-self, leading to chronic inflammation, tissue damage, and diverse clinical manifestations(18). These conditions can affect nearly any organ system. Notable examples include rheumatoid arthritis (RA), systemic lupus erythematosus (SLE), multiple sclerosis (MS), and type 1 diabetes mellitus (T1DM), all of which share immune dysregulation as a core mechanism despite differing clinical features(18, 19).

Cutting-edge AI algorithms are increasingly applied to analyze high-dimensional genetic and molecular data, uncovering new gene-disease associations and immune pathways implicated in autoimmune pathogenesis. For example, AI has improved risk prediction and treatment stratification in SLE and RA by integrating omics data with ML models(20). These tools facilitate early diagnosis, enable patient stratification based on molecular signatures, and predict therapeutic efficacy, thus supporting precision treatment strategies(21).

Drug repurposing is also being explored in immune-mediated diseases; for example, metformin has been evaluated as adjunct therapy in T1DM, with outcomes that can vary by cohort and clinical context(22, 23).While challenges such as data heterogeneity and algorithmic bias remain, AI continues to drive innovation in precision medicine for autoimmune disorders, ultimately improving clinical outcomes and quality of life(20, 21, 24). This review examines recent advances in AI applications in immunotherapy, with a focus on their growing role in cancer treatment and autoimmune diseases management.

**Statement of significance**.

**Table:** 

Heading	Summary
Problem or Issue	Immunotherapy has transformed treatments for cancer and autoimmune diseases, yet major challenges remain in predicting therapeutic response, optimizing drug delivery, and ensuring patient safety, largely due to biological heterogeneity and the complexity of integrating multi-source biomedical data.
What is Already Known	AI has been applied separately in oncology and autoimmune disorders, showing promise in biomarker discovery, patient stratification, drug design, and outcome prediction. However, most reviews address one disease area in isolation, limiting cross-disciplinary insights.
What This Paper Adds	This work bridges oncology and autoimmune immunotherapy, offering a translational and pharmaceutical informatics perspective on AI-driven solutions — including explainable AI, federated learning, and digital twins — to address shared therapeutic challenges and accelerate precision medicine.
Who Would Benefit from the New Knowledge in This Paper	Biomedical informatics researchers, clinical immunologists, pharmaceutical scientists, and healthcare decision-makers involved in developing AI-enabled immunotherapies or optimizing targeted drug delivery for complex immune-mediated diseases.

## AI advances in immunotherapy

**Fig. 1:**
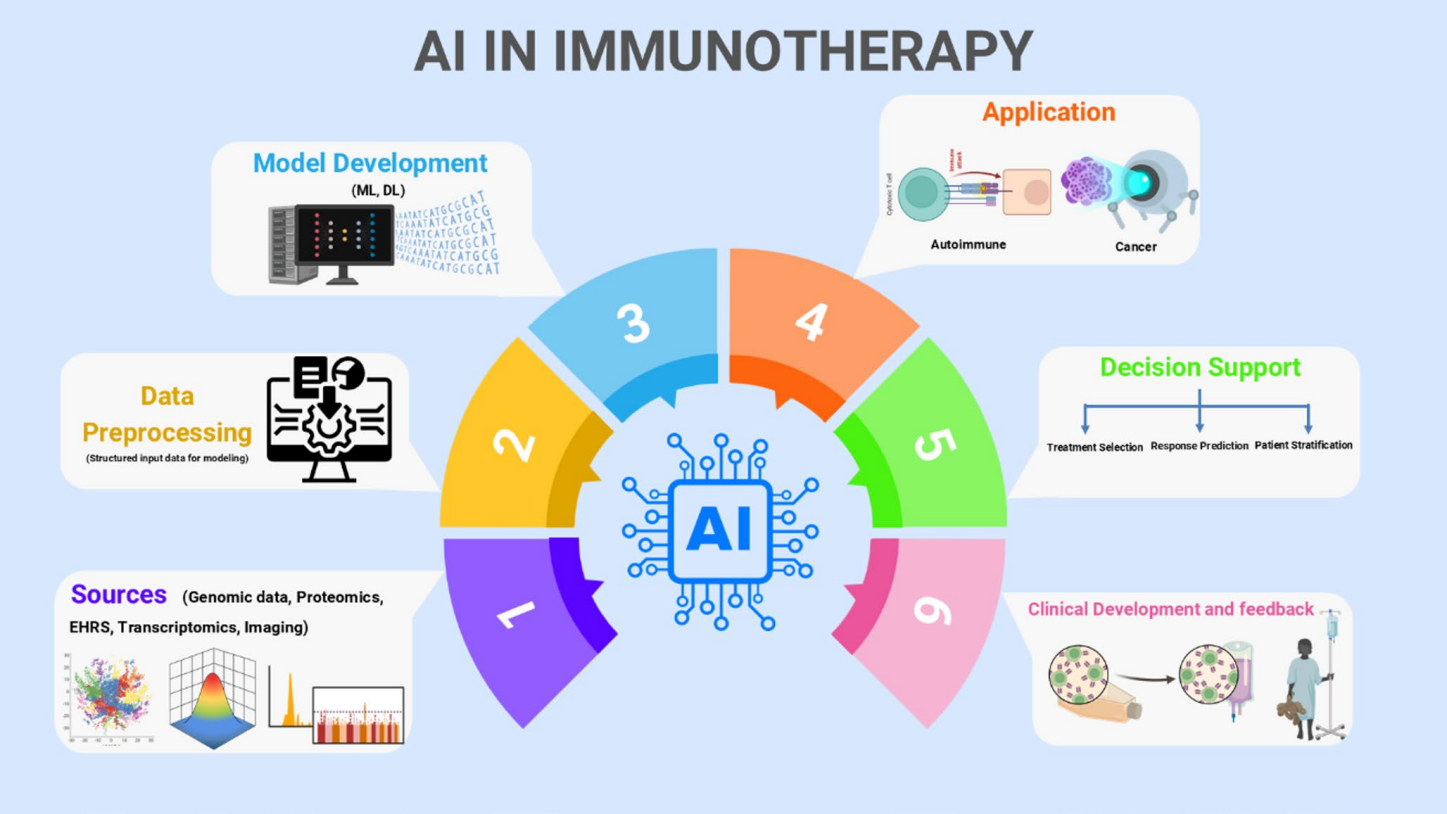
Schematic representation of AI applications in immunotherapy. AI workflows typically begin with the integration of diverse data sources, including genomics, proteomics, transcriptomics, EHRs, and medical imaging. These are subjected to preprocessing to create structured datasets suitable for ML or DL model development. The resulting predictive models are applied to clinical scenarios involving cancer and autoimmune diseases, supporting key tasks including treatment response prediction, biomarker identification, and patient stratification. The feedback loop from clinical deployment informs model refinement and guides ongoing development—**Abbreviations**: *EHR*,* electronic health record; ML*,* machine learning; DL*,* deep learning*

AI-powered imaging biomarkers increasingly enable non-invasive tumor characterization and evaluation of the tumor microenvironment (TME), thereby enhancing patient selection for immunotherapy(25, 26). For instance, in non-small cell lung cancer (NSCLC) and melanoma, AI-derived radiomic features extracted from pre-treatment contrast-enhanced computed tomography (CT) scans have distinguished responders from non-responders. Radiographic heterogeneity- including irregular tumor borders and variable internal density-has been associated with favorable response to immune checkpoint inhibitors (ICIs) in multiple cohorts (25, 27, 28). In digital pathology, AI has revolutionized histological image analysis by enabling automated, high-throughput examination of tumor tissue samples. Generative Adversarial Networks (GANs), have shown remarkable ability to synthesize high-resolution histological images and capture latent image features through unsupervised learning. These advances have improved nuclear segmentation, detection, and domain adaptation, ultimately deepening our understanding of immune cell distribution and tumor architecture within the TME(29, 30). The conceptual framework for applying AI in immunotherapy, including data acquisition, preprocessing, model development, and translational applications, is summarized in Fig. 1.

AI has also played a key role in predicting TMB, a pivotal biomarker associated with responsiveness to immunotherapy. ML algorithms trained to classify TMB levels have enabled more accurate stratification of patients into groups with distinct survival outcomes following ICI treatment(31). Similarly, radiomic signatures derived from CT imaging have been used to characterize immune phenotypes, distinguishing tumor-inflamed and tumor-desert subtypes. High signature scores, reflecting elevated CD8^+^ T-cell infiltration, correlate with improved objective response rates and survival(32, 33). In addition, AI has contributed significantly to post-treatment evaluation. Radiomic-based models comparing baseline and on-treatment imaging, can identify patients with poor therapeutic sensitivity and shortened overall survival. These models quantify dynamic tumor changes, such as alteration in volume, boundary invasion, and spatial heterogeneity, providing early indicators of resistance (31, 34).

Clinical translation is progressing. Although most radiomic signatures for ICI response remain investigational, deep-learning radiomic biomarkers have undergone large, multi-institution validation in real-world cohorts and have also been evaluated using prospective clinical-trial datasets in advanced NSCLC, illustrating movement toward clinically testable decision support for immunotherapy selection(35). In parallel, peer-reviewed regulatory analyses indicate that regulators (including the FDA) have authorized a growing number of AI-enabled medical imaging devices and are refining oversight frameworks for AI software as a medical device, emphasizing clinical validation, generalizability across sites, bias mitigation, and lifecycle/post-deployment monitoring(36). Despite these advances, challenges remain in integrating AI into routine immunotherapy practice. Variability in data quality, limited model interpretability, and the need for large-scale, multicenter validation hinder clinical translation. Addressing these barriers will be essential. Future research should emphasize advanced frameworks such as reinforcement learning for adaptive treatment strategies and federated learning to support secure, privacy-preserving collaboration across institutions(29, 37).

## AI in cancer immunotherapy

AI is redefining the landscape of cancer immunotherapy by processing large-scale, multidimensional biological datasets and discover predictive biomarkers that inform patient-specific responses to treatments, such as immune checkpoint inhibitors (ICIs)(38). One key advancement is the development of platforms such as SCORPIO (Standard Clinical and laboratory featuRes for Prognostication of Immunotherapy Outcomes), a machine-learning model that predicts ICI benefit using widely available clinical variables, including routine blood tests (e.g., complete blood count and standard chemistry panels) and basic patient characteristics. In a large multi-cohort study including 9,745 ICI-treated patients across 21 cancer types, SCORPIO was trained on an internal cohort and validated across independent datasets, showing AUCs in the ~ 0.7–0.76 range for survival/clinical benefit prediction(39). Additionally, AI-based integrative models that combine genomic, proteomic, and imaging data have substantially improved the accuracy of therapeutic outcome predictions. These models not only facilitate the optimization of treatment strategies but also enable the identification of novel immunogenic targets, such as neoantigens. These advancements help address the limitations of conventional biomarkers like PD-L1, which often fail to capture tumor heterogeneity or the dynamics of the immune microenvironment(16, 40). AI has made significant progress in refining predictive biomarker discovery, which is pivotal in enhancing the personalization of cancer care. Furthermore, AI is instrumental in individualizing immunotherapy by evaluating patient response profiles and enabling real-time adjustments to treatment. ML algorithms, particularly those trained on histopathological images and molecular features, have proven effective in predicting immunotherapy responses across various solid tumors(41, 42). For instance, models developed using mass spectrometry-based human leukocyte antigen (HLA) datasets have greatly enhanced the identification of tumor-specific neoantigens, thereby improving immune targeting and overall therapeutic efficacy. Moreover, AI systems enable continuous monitoring of patient responses throughout the treatment course, offering clinicians actionable insights that support early intervention and adaptive therapy planning(33, 43). Despite its transformative potential, the full integration of AI into clinical oncology faces challenges that must be addressed for widespread adoption. These include issues related to data privacy, algorithmic transparency, and the interpretability of complex ML models. As AI models become more advanced, they will require robust regulatory frameworks and transparent standards to ensure their ethical use in clinical practice. Overcoming these barriers will be essential for the successful implementation of AI in routine oncological care. As these challenges are progressively addressed, AI is poised to play an increasingly central role in modern immuno-oncology, supporting precision and personalized cancer treatment(44). An overview of key AI applications in cancer immunotherapy is presented in Table 1.

**Table 1:** AI Applications in Cancer Immunotherapy

AI/ML Model or Algorithm Type	Cancer Type(s)	Data Modalities Used	AI Application Purpose	Performance Metrics	Clinical or Translational Impact	References
Deep Learning (CNNs, RNNs, Transformers)	Non-small cell lung cancer(NSCLC)	Radiomics (CT), histopathology, genomics	PD-L1 prediction, TMB prediction, and ICIs response classification	AUC 0.80–0.95, Sensitivity >85%	Enhanced patient stratification, non-invasive biomarker analysis	(51, 52, 107, 108)
	melanoma	Radiomics, Whole-slide imaging, Genomics	Overall survival prediction, neoantigen discovery, and immune phenotype classification	Accuracy >90%, AUC 0.92	Improved immunotherapy targeting and survival stratification	(56, 65, 135)
	colorectal cancer	Histopathology, Clinical data, Genomics	MSI/dMMR status prediction, TMB prediction	Clinical-grade accuracy, AUC >0.85	Supports FDA biomarker-based therapies	(124, 125, 130)
	Head and Neck Squamous Cell Carcinoma	Transcriptomics, Genomics	Neoantigen prioritization, immune receptor profiling	High precision in immune target selection	Precision targeting of immune checkpoint blockade	(307)
	Bladder Cancer	Histopathology, Clinical Data	Immune landscape profiling, TILs prediction	Accuracy >88%	Better immune characterization for ICIs	(308, 309)
Random Forest/Gradient Boosting Machines	Melanoma	Radiomics (CT), clinical data, and immune profiling	Predict ICI response; classify tumor immune phenotypes	AUC: 0.85–0.90; Accuracy: ~85%	Improves ICI eligibility prediction and patient stratification	(57)
	Breast Cancer	Genomics, proteomics, imaging (radiology)	Drug response prediction; immune subtype classification	Accuracy: ~88%; AUC: 0.91	Optimizes biologics and targeted therapy selection	(310)
	Gastric Cancer	Clinical variables, radiological data, multi-omics	Biomarker discovery, survival outcome modeling	AUC ~0.86, precision ~83%	Enhances prognosis and therapy planning	(311–314)
Support Vector Machines (SVMs)	NSCLC	Radiomics (CT/MRI), histopathology, clinical parameters	Immune phenotype classification, TME analysis, biomarker prediction	Sensitivity >85%, Specificity >80%	Improves immunotherapy stratification; guides biomarker validation	(315, 318)
Reinforcement Learning	CAR-T applicable cancers (hematologic malignancies, solid tumors)	Clinical data, CAR cell characteristics, treatment parameters	Optimization of CAR infusion dosing and scheduling protocols	Early-stage development showing promise for therapy optimization	Potential for improved CAR-T production efficiency and personalized treatment adaptation	(65)
Deep Neural Networks (DNNs)	Melanoma	Peptide sequences, Protein language models (ProtTrans), Multi-window CNN	Neoantigen prediction without requiring specific MHC allele information	AUC: ~0.93; MCC: ~0.53; Accuracy: ~92%	Facilitates accurate neoantigen identification for personalized cancer vaccines and immunotherapies	(319)
	NSCLC	Genomics Clinical Data, PET/CT imaging	Gene mutation prediction (EGFR, KRAS, ALK)	AUC: 0.94	Enables precise targeted therapies and improved mutation-driven treatment planning	(320)
Transformers (BERT, BioBERT)	Head and Neck Cancer, Melanoma	Genomic sequences, transcriptomics	Neoantigen prioritization, immune repertoire profiling	High precision in antigen prediction	Enhanced immune receptor mapping; improved selection of immunotherapy targets	(321)
Multi-omics Integration Models	Lung, Breast, Colorectal Cancers	Genomics, transcriptomics, proteomics, metabolomics, clinical data	Integrated biomarker discovery, response prediction, and TME characterization	AUC >0.90	Comprehensive tumor profiling, precision immunotherapy, and identification of novel therapeutic targets	(322)
Graph Neural Networks (GNNs)	NSCLC	PET/CT radiomics and transcriptomic data	Predict lymph node metastasis; integrate genomic and imaging features	AUC = 0.85	Supports prognosis modeling; enhances multimodal integration for clinical risk assessment	(323)
Convolutional Autoencoders	Melanoma	Dermoscopic images	Skin lesion classification via dimensionality reduction and deep learning	Accuracy ~98–99%	Enhances early diagnosis and classification of melanoma and other skin cancers	(324)
Transfer Learning	Rare Cancers, Heterogeneous Tumors	Imaging	Adapt models to small datasets; improve classification and reduce training time	Accuracy up to 94.2% with small datasets	Enables AI application to rare cancers; improves generalizability with limited data	(325)
Attention-based Multi-task DL	NSCLC	Imaging (CT)	Subtype classification and staging; highlights discriminative features	AUC ~0.96	Improves interpretability and supports clinical decision-making	(326)
Explainable AI (XAI) Methods	All Cancer Types	Multi-omics, imaging, and clinical data	Model interpretability, biomarker importance elucidation	Improved clinical trust; diagnostic accuracy >90%	Enhances clinician acceptance; reveals novel biomarker roles; supports precision treatment	(327– 329)
Capsule Networks	NSCLC (Lung), Colon	Histopathology images	Spatial and morphological feature extraction	Accuracy ~92–99%	Captures hierarchical features; improves diagnostic performance; aids in tumor grading	(330–332)
Hybrid Models (ML + DL Ensembles)	Lung, Colon, Breast Cancers	Histopathological images, clinical records, multimodal data	Tumor subtype classification, biomarker discovery, patient stratification	Accuracy up to 98.9%, AUC ~0.95	Integrates imaging and clinical features; enhances diagnostic accuracy; supports personalized therapy decisions	(333–336)

### AI in predicting response to immune checkpoint inhibitors

ICIs are among the most thoroughly studied and widely utilized immunotherapeutic agents in oncology. They have received FDA approval for treating a variety of cancers, demonstrating significant efficacy in clinical trials(45). In normal physiology, immune checkpoints regulate the immune system by balancing activation and suppression, maintaining immune homeostasis and preventing autoimmunity or excessive inflammation(46). However, cancer cells frequently exploit these regulatory pathways to evade immune surveillance, thereby fostering an immunosuppressive TME that hinders effective immune responses(47). AI plays a crucial role in predicting by learning outcomes-associated patterns from large, heterogenous datasets spanning genomics, transcriptomics, epigenomics, radiomics, and digital pathology, enabling the construction of composite predictive signatures that reflect both tumor-intrinsic features and the immune contexture(33, 48).

PD-L1 remains a critical component of response assessment, and AI-based pipelines—particularly deep learning applied to whole-slide images and ML-based radiomic frameworks—have improved the consistency and scalability of PD-L1-related evaluation across tumor types(49, 50). In NSCLC, AI-assisted whole-slide image analysis has demonstrated improved concordance among pathologists, reaching 90.2% agreement, surpassing traditional manual evaluations. Moreover, multimodal AI systems that integrate pathological and CT-derived features have achieved a validation AUC of 0.80 for predicting response to ICIs, illustrating the value of combining complementary data sources for outcome prediction(51, 52).

Beyond PD-L1, AI models incorporate additional determinants of ICI benefit, including HLA-related features and TMB. ML frameworks that combine transcriptomic, radiogenomic, and digital pathology data have can improve predictive performance by capturing interactions between antigen presentation capacity, tumor heterogeneity and immune infiltration (53, 54). For instances, ML analyses, have linked loss of heterozygosity in HLA genes and intra-tumoral heterogeneity with ICI efficacy in NSCLC(55, 56). Epigenomic signals, including DNA methylation patterns, have also been explored as predictors of immunotherapy response in solid tumors. In parallel, radiomic features reflecting tumor volume, spatial heterogeneity, and edge characteristics, have been associated with clinical outcomes, in melanoma, such features have correlated strongly with overall survival in ICI-treated patients, with AUC values reaching 0.92, supporting non-invasive prediction strategies(56, 57).

**Fig. 2:**
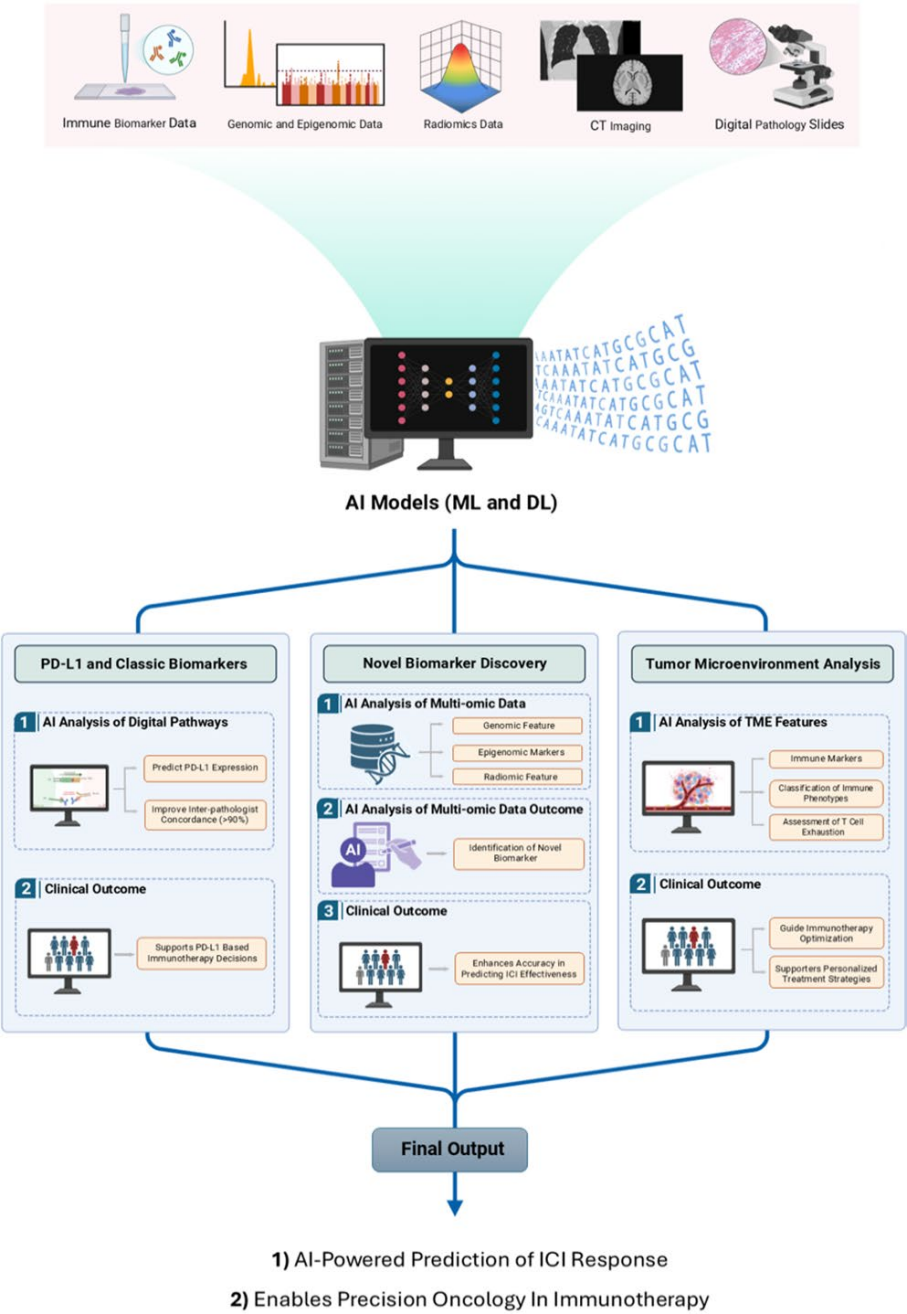
AI-powered framework for predicting immune checkpoint inhibitor (ICI) response and supporting precision immunotherapy. Diverse data types—including immune biomarker profiles, genomic and epigenomic features, radiomic signatures, CT scans, and digital pathology slides—are integrated into ML and DL models. These models facilitate the prediction of PD-L1 expression, the discovery of novel biomarkers, and the characterization of tumor microenvironment (TME) features. Clinical outcomes derived from these analyses include improved PD-L1-based decision-making, enhanced accuracy in forecasting ICI effectiveness, and optimized treatment strategies through immune phenotype classification and T-cell exhaustion profiling. This pipeline supports the broader goal of precision oncology by enabling tailored and timely interventions in immunotherapy

The TME is a complex and dynamic ecosystem composed of diverse immune and stromal cell populations, soluble mediators, and extracellular matrix components that collectively shape immunotherapy responsiveness(58). AI enables systematic characterization of this environment and supports response prediction using immune-related variables such as tumor-infiltrating lymphocytes, systemic inflammatory indices (e.g., neutrophil-to-lymphocyte ratio), and tumor stemness-associated patterns (59, 60). These computational approaches facilitate immune-phenotype classification and help contextualize mechanisms such as T-cell exhaustion and immune evasion. Additionally, integrating emerging platforms (e.g., Raman spectroscopy) with ML has shown promise for assessing treatment effects and refining response prediction in selected settings(61, 62). A schematic overview of the AI-powered workflow for ICI biomarker prediction and treatment optimization is presented in Fig. 2.

### Enhancing CAR-T cell therapy

CAR-T cell therapy is a revolutionary approach that utilizes genetically engineered immune cells designed to recognize and destroy cancer cells. CAR-T cell therapies have achieved remarkable clinical success, particularly in treating hematologic cancers such as B-cell acute lymphoblastic leukemia (ALL), non-Hodgkin B-cell lymphomas, and multiple myeloma, resulting in regulatory approval of several products worldwide(63). However, despite these successes, challenges such as complex manufacturing processes, safety concerns, the difficulty of identifying precise tumor targets, and the regulation of CAR-T cell differentiation and persistence remain substantial barriers to wider adoption(64). AI is transforming CAR-T cell therapy by optimizing each stage, from target discovery to clinical decision-making. By integrating ML and DL, AI addresses manufacturing challenges, enhances therapeutic efficacy, ensures safety, and facilitates personalized treatment strategies. Key applications include optimizing CAR construct designs using structural prediction tools, such as AlphaFold, streamlining manufacturing through predictive algorithms, and identifying biomarkers that predict clinical outcomes and adverse events, such as cytokine release syndrome (CRS)(64, 65). Importantly, AlphaFold was trained primarily on experimentally determined structures of naturally occurring proteins(66); thus, when applied to engineered CAR domains, its outputs should be interpreted as hypothesis-generating structural models that require experimental validation rather than as CAR-specific ground truth(64).These advancements aim to extend CAR-T therapy beyond hematologic malignancies into solid tumors and increase accessibility by lowering production costs(67).

AI plays a vital role in antigen target selection and CAR design by leveraging single-cell RNA sequencing data to identify tumor-specific markers while minimizing off-target effects. For instance, deep generative models have identified myeloid cell targets such as CD86 and CSF1R for acute myeloid leukemia(59, 64). Structural prediction tools optimize the stability and signaling domains of CAR proteins, utilizing ML models trained on thousands of synthetic costimulatory motifs to enhance T-cell activation and longevity. Concurrently, AI-assisted CRISPR-Cas9 genome editing improves precision and reduces off-target mutations, enabling the creation of CAR-T cells tailored for the challenging microenvironment of solid tumors(68, 69). Manufacturing optimization constitutes another critical frontier, where AI predicts ideal culture conditions and monitors cell proliferation via real-time “soft sensors.” Initiatives like AIDPATH utilize digital twin simulations of bioreactor processes, while scheduling algorithms coordinate parallel manufacturing cycles for multiple patients. ML models analyzing multi-omics data from CyTOF and single-cell RNA sequencing predict optimal CD4/CD8 ratios and memory cell proportions in final products, potentially reducing manufacturing failures by up to 30%. These innovations aim to standardize production while preserving flexibility for patient-specific customization(63, 70, 71).

In clinical applications, AI enhances the prediction of treatment responses and the identification of adverse events. Multivariate logistic regression models incorporating lactate dehydrogenase levels and proportions of CAR^+^ regulatory T cells achieve approximately 85% accuracy in forecasting six-month clinical outcomes(72, 73). For toxicity management, ML algorithms analyzing cytokine profiles, including interferon-gamma (IFN-γ) and macrophage inflammatory protein-1 alpha (MIP-1α), demonstrate over 85% sensitivity in predicting severe CRS. Additionally, models trained on PET/CT imaging data facilitate the early detection of relapse. Wearable AI systems enable the remote monitoring of physiological parameters, allowing for timely intervention in cases of side effects such as neurotoxicity(74). As these technologies mature, they hold promise to transform CAR-T therapy into a broadly accessible precision oncology modality capable of treating diverse cancer types with improved safety and efficacy profiles(75).

### AI for neoantigen prediction

Neoantigens, the tumor-specific antigens generated by somatic mutations, represent a promising frontier in cancer immunotherapy. These antigens are expressed exclusively on cancer cells, making them ideal targets for immune-based therapies. However, only a fraction of these neoantigens elicit effective immune responses, necessitating accurate identification of the most immunogenic candidates(76).

AI is a vital tool in neoantigen prediction by addressing the limitations of traditional experimental methods through modeling peptide presentation by the major histocompatibility complex (MHC) and downstream T-cell receptor (TCR) recognition of the resulting peptide–MHC (pMHC) complexes (76, 77). Importantly, high MHC binding affinity mainly reflects antigen presentation likelihood and does not necessarily equate to true immunogenicity, which also requires productive TCR recognition and functional T-cell activation(78). AI-based strategies are revolutionizing neoantigen discovery by integrating multi-omics datasets to identify high-confidence targets. For example, platforms like EasyFuse utilize transcriptomic data to detect cancer-related gene fusions. In contrast, NeoDisc combines genomic, transcriptomic, and proteomic information to pinpoint immunogenic neoantigens more effectively than conventional methods(79). By improving neoantigen prediction and validation, AI accelerates the translation of precision immunotherapies into clinical usage, offering new treatment opportunities for patients with heterogeneous or treatment-resistant cancers(80, 81).

MHC-peptide binding prediction models are generally divided into allele-specific and pan-specific categories. Allele-specific models are trained on peptides corresponding to particular MHC alleles, while pan-specific models predict peptide binding across multiple alleles. These models typically encode MHC and peptide sequences into fixed-length vectors using methods such as one-hot encoding or embeddings(77, 82). Deep neural networks (DNNs) and convolutional neural networks (CNNs) are frequently employed to capture intricate patterns in peptide binding data(83). Notable predictors include NetMHCpan, MHCflurry, and HLAthena, which have enhanced prediction accuracy by incorporating ligand data derived from mass spectrometry(84–86). Although substantial progress has been made in predicting MHC class I (MHC-I) binding, predicting MHC class II (MHC-II) remains challenging due to the heterogeneity in peptide length and binding motifs, compounded by the polymorphic nature of MHC-II molecules(78, 82). However, incorporating MS-derived ligand data has improved the performance of MHC-II predictors, such as CAPTAn and MARIA(87, 88). Additionally, NetMHCIIpan has increased its predictive accuracy by deconvoluting MS-eluted ligand (MS-EL) data and expanding its training dataset to cover a broader range of HLA-II specificities(89, 90). Continued research is needed further to enhance the performance of MHC-II binding prediction models.

Forecasting the recognition of pMHC complexes by TCRs is particularly complex due to the diversity of TCR rearrangements and the spatial complexity of TCR-pMHC interactions(91, 92). Experimental techniques such as multimeric pMHC assays and high-throughput library screens have been developed to study pMHC-TCR binding. Technologies, including lentiviral transfer assays and synthetic peptide display libraries, have expanded the capacity to identify paired pMHC-TCR interactions(93, 94). Despite these advances, accurately predicting neoantigens based on pMHC-TCR binding remains a challenging area that requires further methodological improvements. The identification of tumor antigens and the development of cancer vaccines constitute cutting-edge immunotherapeutic strategies. Neoantigens, derived from somatic mutations unique to individual patients’ tumors, are ideal targets for T-cell-based therapies because they are absent in normal tissues and evade central immune tolerance, allowing for specific recognition by both CD4^+^ and CD8^+^ T-cells. The personalized immunotherapy approach leverages AI to model immune interactions, optimize vaccine design, and efficiently select promising neoantigen candidates(95).

Accordingly, AI-guided mRNA and peptide vaccines have already progressed to phase I clinical trials in various cancers, illustrating AI’s role in accelerating therapeutic development(96, 97). Notably, computational/AI-supported neoantigen ranking is already being translated into personalized neoantigen vaccine trials(98). For example, BioNTech’s individualized mRNA neoantigen vaccine autogene cevumeran has been evaluated as adjuvant therapy in resected pancreatic ductal adenocarcinoma, including in combination with checkpoint blockade, demonstrating feasibility and induction of neoantigen-specific T-cell responses in a subset of patients(99). In parallel, Moderna’s individualized neoantigen therapy mRNA-4359 has been tested clinically in combination regimens (e.g., with pembrolizumab), as evaluated in resected melanoma, providing a concrete example of personalized neoantigen vaccine development in trials(96). Adjuvants further enhance vaccine efficacy by amplifying immune responses, and AI facilitates their design through virtual screening, property prediction, and drug repurposing efforts. For example, AI has identified CXCL12 inhibitors with potential as potent adjuvants(100, 101). By integrating genomics, bioinformatics, and machine learning, these innovations enable the precise selection of neoantigens and the formulation of vaccines, offering tailored solutions for tumors resistant to conventional therapies while addressing challenges such as immune suppression and tumor heterogeneity(102).

### AI for direct prediction of immunotherapy responses

AI in cancer immunotherapy enables the detection of subtle phenotypic differences and generates detailed data that augment human assessment (103). AI algorithms excel in processing complex medical information, including imaging and large-scale datasets, to accurately and consistently forecast patient responses to ICB therapies(104). This capability supports the implementation of personalized treatment plans, ultimately improving clinical outcomes for cancer patients. In the realm of medical imaging, AI enhances radiological workflows by integrating high-dimensional data from modalities such as CT and magnetic resonance imaging (MRI)(105). AI-powered radiomic models non-invasively analyze tumor morphology and TME features, offering insights that outperform traditional imaging interpretation(106). These models have been particularly effective in predicting immunotherapy responses in cancers such as NSCLC, melanoma, and esophageal carcinoma. Radiomic biomarkers identified by AI also show correlations with genomic indicators, such as TMB, refining response prediction accuracy(107, 108).

**Fig. 3:**
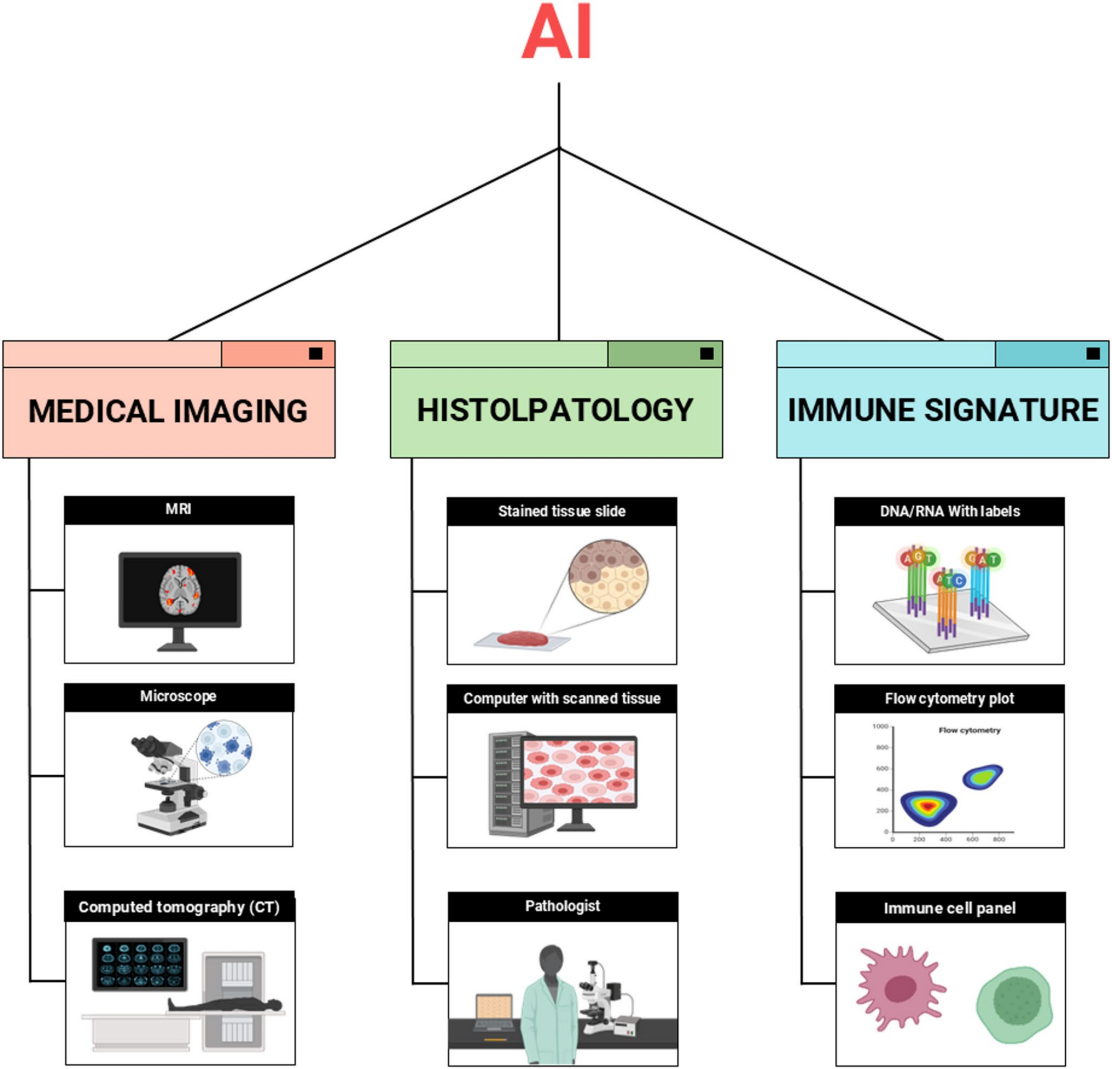
Core data modalities integrated by AI for direct prediction of immunotherapy response. AI models utilize multimodal sources including (1) medical imaging (e.g., CT, MRI, microscopy) for radiomic feature extraction (2), histopathological analysis of whole-slide tissue sections to identify immune infiltration and morphological markers, and (3) immune profiling using genomic assays, flow cytometry, and immune cell panels. These datasets enable AI to detect clinically relevant tumor and immune signatures, enhancing the precision of response prediction in cancer immunotherapy

Histopathology, the gold standard for cancer diagnosis, has similarly been transformed by AI. ML and DL applied to whole-slide histological images enable the precise identification and classification of cellular structures and protein expression patterns(109, 110). These AI-enhanced histopathological analyses enhance the prediction of immunotherapy responses by identifying abnormal morphological features and immune-related molecular markers within the tumor microenvironment. Such techniques facilitate more efficient patient selection for immunotherapy, potentially extending benefits to a broader patient population(111, 112). Figure 3 summarizes the major AI-integrated data domains, including medical imaging, histopathology, and immune signatures, used to predict immunotherapy outcomes directly. Beyond imaging and pathology, AI integrates multi-omics data to construct comprehensive predictive models. These incorporate a variety of biomarkers such as PD-L1 expression, TMB, tumor-infiltrating lymphocytes (TILs), and specific genetic mutations, thereby enhancing the reliability of immunotherapy response predictions. The integration of diverse data types enables a more nuanced characterization of tumors, advancing precision medicine and individualized treatment planning(113, 114).

Recent innovations include AI platforms like SCORPIO, which leverage routine clinical data, i.e., blood laboratory results and patient demographics, to predict responses to ICIs. SCORPIO surpasses current FDA-approved biomarkers in predictive performance with a faster, cost-effective, and widely accessible tool for guiding immunotherapy decisions. These predictive capabilities hold promise for improving treatment equity and reducing healthcare costs globally(39, 115). Overall, AI’s contributions to cancer immunotherapy encompass detailed phenotypic profiling, advanced image analysis, histopathological interpretation, and the integration of multi-omics datasets, collectively enhancing the therapeutic response prediction, optimizing patient stratification, and supporting personalized immunotherapy to ensure precision oncology(16).

### Predictive biomarkers and immunotherapy

AI models, particularly those employing ML and DL techniques, have demonstrated robust discriminative performance (e.g., AUC and sensitivity/specificity profiles) in detecting key immunotherapy-related biomarkers, such as PD-L1 expression, TMB, and microsatellite instability (MSI). Methods like Random Forest algorithms improve patient stratification for ICIs, thereby enhancing the personalization of treatments(116, 117). DL architectures, including convolutional neural networks (CNNs) and recurrent neural networks (RNNs), effectively process large-scale spatial and sequential datasets, enabling the identification of complex biomarkers, such as gene expression profiles predictive of ICI responsiveness. These AI approaches exhibit strong reliability, with AUC values ranging from 0.70 to 0.95, outperforming traditional, manual biomarker detection methods that tend to be slower, less precise, and less reproducible. Furthermore, AI’s capacity to integrate multi-omics data has revolutionized biomarker identification by uncovering intricate patterns that conventional techniques often miss(118, 119).

Accurately detecting the expression status of tumor molecular pathological markers is essential before initiating clinical immunotherapy, as these markers guide treatment decisions and predict patient responses(120). For example, pembrolizumab (Keytruda) has gained FDA approval as a first-line treatment for patients with MSI-high (MSI-H) metastatic colorectal cancer, underscoring the clinical importance of such biomarkers. In addition to MSI, other genomic features, including TMB, homologous recombination deficiency (HRD), and whole-genome duplication carry significant predictive value(120–122). Consequently, there is a growing demand for rapid and cost-effective biomarker detection methods, including approaches based on routine hematoxylin and eosin (H&E)-stained histopathology images, quantitative PCR (qPCR), immunohistochemistry (IHC), and next-generation sequencing (NGS), many of which do not require additional tissue samples(38).

While MSI testing is clinically valuable, it is not always performed due to the need for supplementary genetic or immunohistochemical assays. Advances in DL have enabled the direct prediction of MSI status from widely available H&E-stained histology slides, with model performance benchmarked against clinically determined MSI reference standards—typically PCR-based MSI testing and/or IHC-defined mismatch repair deficiency (dMMR)—and with formalin-fixed, paraffin-embedded (FFPE) slides demonstrating higher predictive accuracy than snap-frozen specimens(117, 123). Additionally, circulating tumor DNA (ctDNA) analysis has been used to predict MSI status in endometrial cancer patients, thereby supporting informed decision-making for immunotherapy. Large multicenter studies using DL models trained on whole-slide images of colorectal tumors have achieved clinical-grade accuracy in detecting MSI and mismatch repair deficiency (dMMR), surpassing even the accuracy of expert pathologists. Nevertheless, challenges remain in generalizing these models across diverse datasets, partly due to variability in slide preparation and scanning protocols(124, 125). AI has also been effectively used to predict PD-L1 expression. ML algorithms, including random forest classifiers and generative adversarial networks, have been used to quantitatively assess PD-L1 expression in tumor cells with high agreement compared to pathologists’ manual scoring(126). Fully automated CNNs have further enhanced tumor cell detection and PD-L1 tumor proportion scoring (TPS), improving diagnostic consistency and efficiency, particularly among less experienced pathologists. These AI-enabled methods hold promise for standardizing and accelerating PD-L1 evaluation in clinical practice(127, 128).

TMB is another pivotal biomarker associated with immunotherapy response, as elevated TMB correlates with a higher neoantigen load and stronger immune recognition. DL models such as Image2TMB have been developed to predict TMB status directly from H&E slides, potentially replacing costly and time-intensive whole-exome sequencing(129, 130). Moreover, multimodal DL approaches that integrate histopathological images with clinical data have improved the accuracy of TMB prediction in cancers such as colorectal carcinoma. Clinical variables, such as tumor stage and patient age, have also been shown to have significant associations with TMB status, emphasizing the benefit of combining molecular and clinical information to tailor personalized immunotherapy strategies(130, 131).

### AI for antibody prediction

AI is accelerating antibody discovery in cancer immunotherapy by enabling faster and more cost-efficient design of therapeutic antibodies(132). Advanced ML and DL models can predict antibody–antigen interactions, optimize binding affinity, and evaluate developability—key determinants of efficacy, safety, and manufacturability(133). For example, AI-driven platforms such as ADCNet integrate protein and small-molecule information to predict antibody–drug conjugate activity. Generative approaches, including variational autoencoders and language models, are increasingly used to propose novel antibody sequences, often with an emphasis on complementarity-determining regions (CDRs) that govern binding specificity(134, 135). In this review, “ImmunoNet” (mentioned later) is used as a hypothetical placeholder for a multimodal AI framework unless explicitly linked to a specific published model. Sequence design and affinity maturation. Recent progress in AI and DL has substantially advanced antibody discovery, particularly in target binding screening and affinity maturation. While high-throughput sequencing(HTS) and display libraries can explore vast antibody sequence spaces, much of the potential diversity remains experimentally inaccessible(136, 137).

AI approaches, including neural networks and generative algorithms, have therefore been integrated with experimental workflows to predict and enhance antibody sequences, improving affinity and specificity. For instance, ensemble neural networks can outperform individual models in predicting sequence enrichment during phage display panning, and gradient-based frameworks such as Ens-Grad propose sequence modifications that yield superior binding compared with training-set antibodies. Deep mutational scanning paired with CNNs further supports rational design of combinatorial libraries, reducing the search space and improving discovery of high-affinity binders(137, 138). AI-driven affinity maturation can also streamline iterative optimization by forecasting productive mutations and prioritizing candidates using confidence estimates, thereby improving efficiency early discovery efficiency(139).

Recent advances include LSTM-based deep generative models with downstream ranking (e.g., linear discriminant analysis) to generate and prioritize variants with progressively enhanced affinity and specificity, frequently surpassing frequency-only screening strategies(140). Despite these gains, distribution shift and limited negative/failed examples remain practical constraints, underscoring the importance of prospective, experiment-in-the-loop validation when deploying AI-guided sequence optimization(139). Structure prediction and antibody-antigen modeling. Recent advancements in DL have reduced reliance on labor-intensive and expensive experimental structure determination (e.g., crystallography, NMR, and cryo-electron microscopy).

However, antibody structure prediction remains challenging because antibodies exhibit high conformational variability, particularly in the CDR H3 loop, and general protein structure predictors can underperform in these regions(66, 141). Moreover, the limited availability and diversity of high-quality experimentally resolved antibody structures (especially antibody–antigen complexes) remains a persistent bottleneck for training, benchmarking, and generalizing antibody-specific structural models. To address these challenges, antibody-focused models can be grouped by function. For CDR-focused loop modeling, DeepH3 uses deep residual neural networks with geometric features to improve prediction of CDR H3 conformations, outperforming earlier approaches in benchmark evaluations(142, 143). For full variable-domain modeling (including side chains), DeepAb integrates unsupervised representation learning and attention mechanisms to enhance interpretability and accuracy, while DeepSCAb incorporates side-chain geometry prediction, which further refines structural forecasts(144, 145).

However, some approaches remain multi-step and lack equivariance, which can limit speed and flexibility. For fast, end-to-end CDR loop prediction with scalable throughput, ABlooper applies E(n)-equivariant graph neural networks and provides reliable quality assessment, making it suitable for large-scale repertoire analyses(146, 147). For nanobody-specific modeling, NanoNet directly predicts 3D backbone coordinates, enabling rapid, high-throughput structure prediction useful for antibody–nanobody docking and epitope mapping(148). For integrated antibody–antigen pipelines, AbAdapt combines antibody and antigen modeling, epitope/paratope prediction, and rigid docking, leveraging state-of-the-art tools such as AlphaFold2 to accelerate antibody–antigen interaction studies((149, 150). AI and ML are also transforming prediction and optimization of key pharmaceutical properties in therapeutic antibodies, addressing specificity, immunogenicity, aggregation, solubility, viscosity, and pharmacokinetics. Traditional experimental approaches for developability screening are limited by material requirements and throughput, complicating early-stage triage(151, 152).

AI-driven techniques, including DL and protein language models, now support rapid prediction of these properties directly from antibody sequences. Examples include bidirectional LSTM networks and transformer-based tools such as BioPhi for evaluating antibody “nativeness” and humanization needs, as well as solPredict and DeepSCM for quantitative prediction of solubility and viscosity using transfer learning and CNN-based models(153–156). These approaches enable earlier elimination of candidates with unfavorable developability profiles, accelerating progression to downstream validation.

Beyond developability, AI models can support antibody optimization by simultaneously considering binding affinity and “naturalness”—which is associated with both key efficacy and safety. Deep contextual language models trained on affinity datasets can predict binding of novel variants and optimize functional and biophysical properties, outperforming purely random mutagenesis plus screening workflows(157, 158). AI can also integrate structural and in vitro assay data to predict pharmacokinetic behavior, identifying features such as isoelectric point and poly-specificity that influence antibody clearance. As more high-quality data becomes available, these ML methods are expected to continue improving, facilitating rational design of antibodies with superior therapeutic profiles and paving the way for safer, more effective biologic drugs (158, 159). A summary of AI applications in the diagnosis, treatment planning, and management of autoimmune diseases is provided in Table 2.

**Table 2:** AI Applications in Autoimmune Disease Management

AI/ML Model or Algorithm Type	Autoimmune Disease(s)	Data Sources	AI Application Purpose	Performance Metrics	Clinical or Translational Impact	References
CNNs	RA, Psoriasis, SLE	Radiographic (RA), Dermoscopic (Psoriasis), and Skin Images (SLE)	Automated diagnosis, lesion classification, inflammation assessment	AUC: 0.90–0.98; Accuracy: 91–96%	Enables early detection and image-based disease grading; supports differential diagnosis	(237, 337–339)
ANNs	RA, SLE	Clinical, lab, and serological data	Predict treatment outcomes, disease classification, and remission likelihood	AUC ~ 0.78–0.92; F1-score up to 0.91	Supports personalized therapy and disease severity stratification	(340, 341)
SVMs	MS	Blood biomarkers (Vit D3, B12, Se)	Diagnostic support	Sensitivity ~ 98.98%, Accuracy ~ 98.89%	Non-invasive, cost-effective diagnostic aid	(342)
XAI	RA, SLE	Multi-omics, HER, SNP	Biomarker discovery, transparent decision support	AUC > 90%	Enhances interpretability; enables personalized clinical decisions	(343, 344)
Random Forests & Gradient Boosting	RA	clinical/lab data	Predict biologic therapy response	AUC ~ 0.64–0.75	AUC ~ 0.64–0.75	(345, 346)
DL Models (DNNs, RNNs)	RA, MS, T1D Psoriasis	Imaging, genomics, clinical data	Diagnosis, disease progression prediction, patient stratification	AUC 0.80–0.92	Supports early detection and precision medicine; enables personalized interventions	(249, 347–352)
NLP	SLE, RA	EHR, clinical notes	Information extraction, risk assessment	Boosted diagnosis accuracy	Enhances structured data extraction from unstructured notes	(353–355)
Reinforcement Learning	MS	Force plate data (balance metrics)	Disease severity assessment and modeling	Qualitative improvement in balance modeling	Aids in quantifying MS progression and motor control	(356)
Decision Trees & Ensemble Methods	T1D, RA	EHR, biometrics, CGM glucose logs, cytokine data	Early diagnosis, complication prediction (e.g., hypoglycemia, RA activity)	Accuracy 78–93%	Supports timely diagnosis, predicts complications like hypoglycemia or disease flare	(357–359)
Transfer Learning	RA	Artificially augmented sparse datasets	Synovitis classification for disease monitoring	AUC 0.73–0.99	Enables AI application in data-scarce imaging scenarios	(360)
Digital Twin Models	T1D, MS	Multi-omics + clinical time series	In silico therapy simulation and precision dosing	Under validation; high simulation accuracy	Enables pre-trial prediction of individualized treatment responses and disease progression	(285, 361–363)

## AI Applications in autoimmune disease diagnosis and therapy

The immune system is essential for protecting the body against foreign agents like bacteria, viruses and toxins. Upon detecting these invaders, the immune system mobilizes various defense cells to eliminate the threat. Normally, the immune system distinguishes between foreign entities and the body’s own cells. However, when this balance is disrupted and the immune system attacks healthy self-cells, autoimmune disorders occur(18). These disorders can affect a wide range of organs and tissues, including blood vessels, connective tissue, endocrine glands (e.g., thyroid and pancreas), muscles, joints, skin, and red blood cells. To date, over 80 autoimmune diseases have been identified, with more likely undiscovered. Contributing factors include genetics, diet, infections, and chemical exposures. Diagnosing autoimmune diseases remains challenging due to the diversity of symptoms, which vary based on the affected organ or tissue and often include fatigue, redness, swelling, warmth, and pain(160).

Given the complexity of their clinical manifestations and underlying pathophysiology, autoimmune diseases present significant diagnostic and therapeutic challenges. AI has shown transformative potential in this area, particularly in improving diagnostic accuracy, enabling personalized treatment strategies, and providing real-time disease monitoring(161).

AI algorithms can process vast and diverse datasets, including EHRs, laboratory results, medical imaging, and genomic data(21). By identifying subtle patterns within these datasets, AI supports earlier diagnosis, better disease prognosis and optimized therapeutic approaches(162).

For example, ML-based EHR phenotyping has been used to identify SLE cohorts using structured codes and laboratory data, with NLP-derived narrative features evaluated as additional signals(163). Similarly, AI analysis of OCT (including AS-OCT) can quantify intraocular inflammatory activity and assist uveitis grading, demonstrated correlation with Standardization of Uveitis Nomenclature (SUN) clinical grades and supporting earlier and more standardized assessment(164, 165). Despite its promise, challenges related to model development, data quality, and personalized treatment persist. Future progress is expected to rely on improved data integration and the refinement of AI algorithm, ultimately enhancing the diagnostic precision, treatment effectiveness, and disease monitoring. The application of AI in autoimmune disease research has grown considerably with advancements in AI technologies over the past two decades(162, 166). In the remainder of this section, we cover diagnostic applications, therapeutic applications (including drug discovery and treatment planning), and disease-specific examples in rheumatoid arthritis and type 1 diabetes.

### Role of AI in diagnosing autoimmune disorders

AI, along with its associated models and algorithms, has become an invaluable asset in diagnosing autoimmune diseases, particularly in areas such as image analysis, laboratory data interpretation, and clinical decision support systems(167, 168). Furthermore, integrating AI with EHRs, wearable technologies, and patient-generated data has expanded the scope and precision of disease evaluation(169). AI-based image analysis has shown significant promise in diagnosing autoimmune conditions, particularly RA and Immune-mediated dermatological diseases. In RA, ML and DL models can evaluate radiographs and ultrasound images to detect and quantify pathological features such as joint erosions and synovial hypertrophy, supporting earlier recognition and objective assessment of structural damage (170, 171). Similarly, AI models can analyze images of skin lesions in autoimmune dermatological diseases, recognizing characteristic patterns and estimating the prevalence of these diseases. DL models trained on large dermoscopic or histopathological image repositories facilitate automatic detection of disease-specific features, assisting dermatologists in diagnosis and personalized treatment planning(172). Representative performance has been reported for clinical image classification; for example, an EfficientNet-B4–based dermatology assistant achieved 91.4% sensitivity and 95.48% specificity for psoriasis when classifying clinical images among psoriasis, eczema/atopic dermatitis, and healthy skin in a labeled dataset(173). AI also plays a pivotal role in analyzing laboratory data for the diagnosis of autoimmune diseases, particularly neurological disorders such as MS and neuromyelitis optica (NMO). AI-driven models analyze MRI scans to detect and quantify lesions in the brain and spinal cord, utilizing ML to discern patterns and structural abnormalities that differentiate among autoimmune neurological conditions(174–177). Additionally, AI-based evaluation of serological markers, including autoantibody profiles, supports diagnosis and disease monitoring. For example, AI models that integrate patient-specific autoantibody data and clinical parameters can predict disease activity and risk of organ involvement in SLE, thereby assisting clinicians with personalized prognoses and decision-making(178, 179). AI-powered clinical decision support systems provide crucial assistance to healthcare providers in diagnosing autoimmune disorders. By synthesizing patient data, scientific literature, and AI-derived analyses, these systems generate evidence-based recommendations to improve diagnostic accuracy(180).

These systems process EHR information, which includes patient history, symptoms, and laboratory results, to propose diagnostic hypotheses and suggest appropriate tests. They also assist in differential diagnosis. Additionally, these AI systems analyze longitudinal patient data, which encompasses disease progression, treatment responses, and adverse effects, to improve and refine diagnostic workflows(181). Continuous learning from incoming data enables these platforms to dynamically update recommendations, contributing to the timely and individualized diagnosis of autoimmune diseases(182, 183).

The fusion of AI with EHRs, wearable devices, and patient-reported data enables a comprehensive assessment of autoimmune diseases. AI algorithms can extract hidden trends, correlations, and risk factors from EHR datasets(184, 185). Using natural language processing (NLP), AI can extract structured variables from unstructured clinical notes to support accurate diagnosis and prognosis. Wearable technologies, such as smartwatches and biosensors, continuously capture physiological data, including heart rate, temperature, and activity levels(186). AI analyzes these data streams in context to detect disease flares, predict symptom exacerbations, and optimize therapeutic interventions. Patient-generated inputs—such as symptom diaries and self-reported outcomes—can also be processed by AI to enhance disease monitoring and diagnostic accuracy(187, 188). Applications of AI in autoimmune disease diagnosis span image interpretation, laboratory data analysis, and clinical decision support systems(189). These applications demonstrate AI’s potential to enhance diagnostic precision, promote personalized medical care, and facilitate comprehensive disease evaluation (190, 191). Algorithmic bias can arise when protected groups are underrepresented in training data (minority bias) or when deployment populations differ from training data (training–serving skew), reducing accuracy in those groups; therefore, subgroup performance reporting and equity-focused evaluation before and during deployment are essential(192). As AI technologies advance, their integration into healthcare systems is expected to improve the efficiency and effectiveness of autoimmune disease diagnosis, ultimately leading to better patient outcomes(191).

### AI-driven drug discovery for autoimmune diseases

AI is reshaping drug development and precision medicine for autoimmune disorders, which are characterized by considerable heterogeneity in clinical symptoms and underlying biological mechanisms(193). While contemporary treatment strategies are effective for some patients, they often fail to address individual variability, resulting in inconsistent therapeutic outcomes and unmet patient needs. AI-powered approaches, leveraging extensive multi-omics datasets and sophisticated computational techniques, are enabling deeper mechanistic insight into these complex diseases and facilitating the design of more personalized treatment strategies(193, 194). By integrating high-throughput omics technologies with AI and ML, detailed molecular profiles can be generated from large patient cohorts. Analysis of these profiles using ML algorithms facilitates patient stratification into subgroups that share common biological signatures(195). Through clustering patients based on molecular and clinical characteristics, AI models can delineate distinct endotypes within autoimmune diseases, each characterized by specific dysregulated pathways (e.g., interferon signaling or acute-phase responses). This stratification is essential for customizing treatments according to the unique pathophysiological features of patient subgroups, moving beyond the traditional one-size-fits-all approach(21, 195).

AI-driven modeling has also advanced the discovery of therapeutic targets in autoimmune diseases by mapping genes and proteins that are differentially expressed in specific patient clusters and applying pathway enrichment and network analyses, researchers can identify master regulators and driver mutations instrumental in disease progression. These insights facilitate the selection of highly relevant and druggable targets, accelerating the development of more effective and safer therapies(196–198). In the realm of drug development, AI is revolutionizing the design and optimization of novel drug candidates. Predictive models simulate interactions between millions of potential compounds and specific molecular targets, dramatically reducing the time and cost associated with conventional experimental methods(199, 200). A real-world illustration is AI-enabled drug repurposing, exemplified by BenevolentAI’s knowledge-graph approach that prioritized baricitinib for immune-mediated indications, highlighting how AI can accelerate hypothesis generation and candidate prioritization for downstream validation(201, 202). Moreover, AI-enabled “virtual patient” models enable in-silico assessment of drug efficacy and safety, allowing researchers to predict responses in individual patients or subgroups prior to clinical trials. However, these “virtual patient”/digital-twin approaches remain an emerging capability; their predictive performance and clinical utility require iterative experimental and clinical validation and are still being established across autoimmune indications(203). This computational precision medicine approach holds great promise for delivering targeted therapies with improved benefit-risk profiles(204, 205). Importantly, the quality and interoperability of datasets used to train AI models are critical, alongside the need for standardized methodologies and well-characterized, sufficiently large patient cohorts. Ethical considerations—including data privacy, algorithmic bias, and transparency—must be rigorously addressed to ensure the equitable and trustworthy deployment of AI-based solutions(188, 206).

Looking forward, ongoing advancements in AI technologies, such as DL and digital twin modeling, are expected further to accelerate the transition to precision medicine in autoimmune disorders. These tools will enhance the monitoring of disease progression, prediction of treatment responses, and the real-time dynamic adjustment of therapeutic regimens(207, 208). The identification and validation of strong molecular and digital biomarkers will be crucial for enabling more precise patient stratification and disease monitoring, ultimately improving outcomes and quality of life for those affected by autoimmune diseases(162).

### AI in treatment planning and management

AI is rapidly reshaping treatment planning and management for autoimmune diseases, which are characterized by complex pathophysiology and substantial variability in clinical presentation and therapeutic response(209). Conventional treatment approaches have primarily relied on empirical methods, often resulting in suboptimal outcomes, delayed interventions, and an increased risk of irreversible organ damage. AI-driven techniques, especially those employing ML and DL, can integrate and analyze extensive multidimensional datasets—spanning genetic, proteomic, imaging, and clinical data—to uncover novel biomarkers, stratify patients by risk, and predict disease progression with unprecedented precision(161, 193). For instance, ImmunoNet (a published DL framework) utilizes CNNs and multi-layer perceptrons (MLPs) to synthesize multi-omics and clinical information for disease classification, with up to 98% accuracy reported in the original study; however, this performance should be interpreted in the context of the study’s validation design and cohort characteristics, and independent external validation in diverse populations is needed to confirm generalizability (210).

One of AI’s most impactful contributions in autoimmune disorders lies in facilitating personalized treatment planning. By leveraging patient-specific data such as genetic profiles, molecular signatures, and longitudinal clinical records, AI algorithms like ImmunoNet can predict individual responses to therapies, including biologics, corticosteroids, and disease-modifying antirheumatic drugs (DMARDs), thereby reducing reliance on trial-and-error approaches(211, 212). In diseases such as RA and SLE, AI models can analyze immune cell phenotypes and genetic polymorphisms to stratify patients for targeted biologic therapies, enhancing efficacy while minimizing adverse effects. These systems also incorporate real-time data from wearable devices and EHRs to adjust treatment regimens, as exemplified in MS management dynamically. Such innovations are shifting clinical practice away from one-size-fits-all care toward truly individualized care(213–215).

Computational intelligence methods have become indispensable tools for biomarker discovery, especially in complex autoimmune diseases. These techniques employ advanced algorithms, including fuzzy logic, genetic algorithms, artificial neural networks (ANNs), support vector machines (SVMs), and clustering methods, to analyze large, heterogeneous biological datasets derived from genomics, proteomics, metabolomics, and clinical records(216). By integrating diverse data sources, computational intelligence can reveal hidden patterns and associations that may identify potential biomarkers linked to disease onset and progression. These approaches also support candidate biomarker validation through cross-validation and the development of predictive models to inform personalized medicine, treatment, and prevention strategies(217).

In addition, modern deep-learning architectures—particularly transformer-based multimodal models that integrate medical imaging with routinely collected EHR variables (e.g., diagnostic codes, medications, and laboratory tests)—are increasingly used to capture complex temporal dependencies and improve representation learning in biomedical prediction tasks(218).

AI is revolutionizing disease monitoring and clinical decision support through continuous analysis of clinical, laboratory, and imaging data. Platforms such as ImmunoNet utilize XAI techniques to provide transparent insights into disease activity, enabling early detection of flares and complications. Remote monitoring tools combined with AI algorithms analyze data streams from wearable devices to assess disease status outside clinical settings, thereby enhancing patient engagement and facilitating proactive interventions. This capability is particularly vital for relapsing-remitting diseases, such as SLE, where timely adjustments to immunomodulatory therapies or corticosteroids can prevent irreversible damage(219, 220).

Despite these advances, challenges remain, including data privacy concerns, algorithmic bias, and the opaque “black box” nature of some AI models. Federated learning approaches, implemented in frameworks such as ImmunoNet, help address privacy issues by decentralizing data processing, while XAI methods enhance transparency to support clinical adoption(221). Interdisciplinary collaboration among clinicians, data scientists, and regulatory bodies is crucial for establishing ethical guidelines and robust validation frameworks. As these hurdles are overcome, AI is poised to transform autoimmune disease management, delivering precise, patient-centered care that enhances long-term outcomes and quality of life(219, 222, 223).

### AI in rheumatoid arthritis

**Fig. 4:**
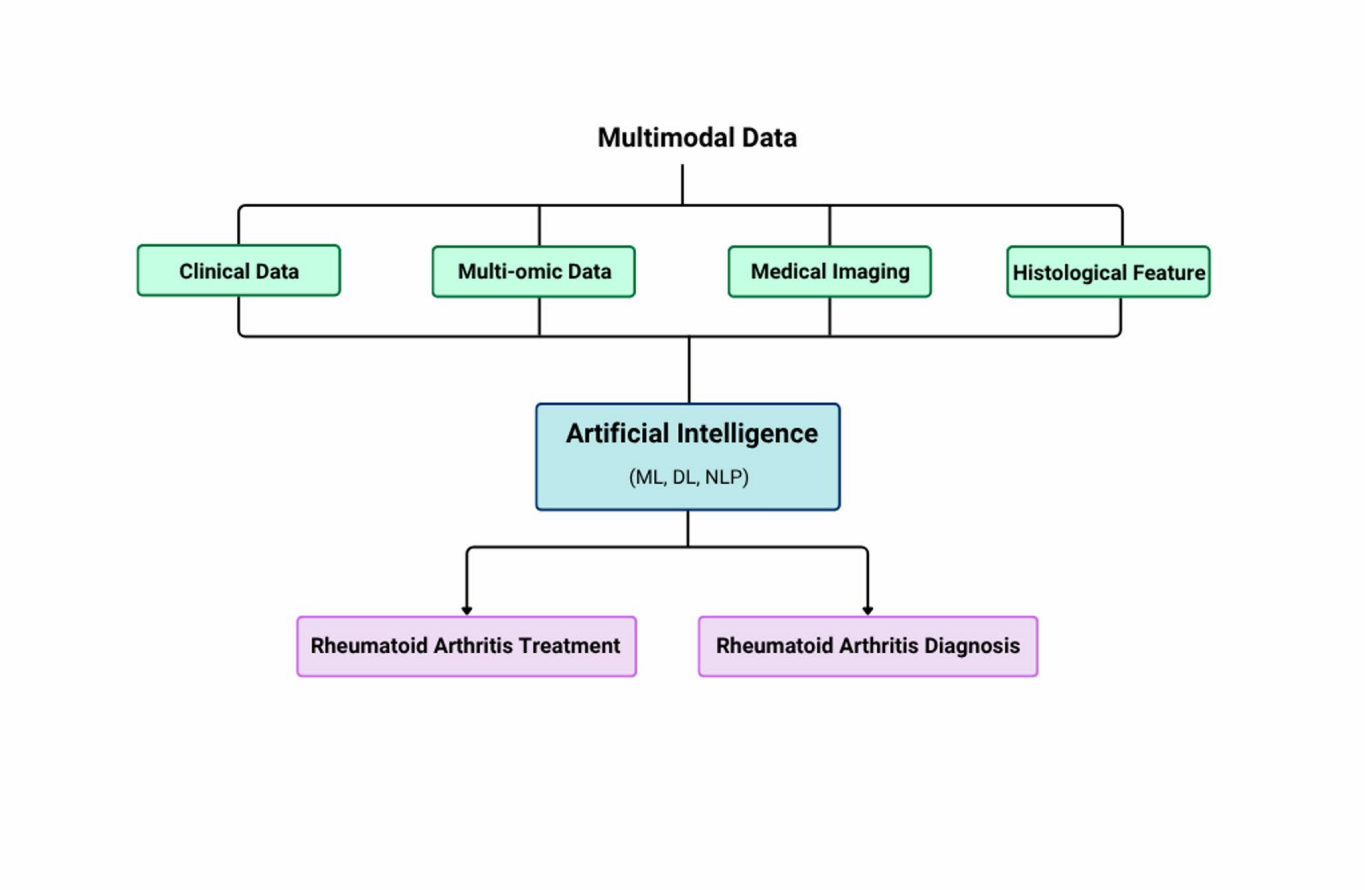
Schematic representation of the AI-enabled pipeline for rheumatoid arthritis (RA) management. Multimodal data, including clinical records, multi-omics profiles, medical imaging, and histopathological features, are processed using ML, DL, and natural language processing (NLP) algorithms. These AI models support both diagnosis and personalized treatment planning, thereby contributing to the advancement of precision medicine in RA

RA is a chronic autoimmune condition primarily targeting synovial joints, resulting in inflammation and potential joint damage(224). Affecting about 1% of the global population, RA is more common among women than men. Over the past five years, AI has made significant advancements in the field of rheumatology(225). AI has rapidly become a transformative tool in managing RA, a complex autoimmune disease characterized by wide variability in clinical symptoms and treatment responses(226). The inconsistent outcomes seen with advanced therapies, such as bDMARDs, highlight the urgent need for precision medicine approaches(227). Leveraging large and diverse datasets, AI methodologies now provide the capability to move beyond empirical treatment decisions, offering personalized and more effective interventions that can enhance patient prognosis and quality of life. AI also facilitates the identification and modeling of factors influencing treatment response. By integrating extensive clinical datasets, electronic health records, and multi-omics data, AI algorithms uncover subtle patterns and associations often missed by conventional analyses(193, 226, 227). For example, XAI methods, such as Shapley additive explanations (SHAP), have clarified the relative impact of clinical features on predicting remission among RA patients treated with biologics(228). These insights support individualized treatment planning and facilitate the discovery of novel biomarkers and therapeutic targets, thereby advancing rheumatology research(229). As illustrated in Fig. 4, multimodal data—including clinical, imaging, histopathological, and molecular profiles—are integrated through AI methodologies such as ML, DL, and NLP. These models facilitate early diagnosis, optimize therapeutic strategies, and enable personalized RA care through accurate risk prediction and outcome modeling. The integration of AI in rheumatology is part of a broader digital transformation in healthcare, including electronic health records, telemedicine, wearable devices, and mobile health applications(230). AI’s strength lies in processing and interpreting complex, large-scale data, enabling the identification of intricate patterns and relationships that exceed human capacity(231). In RA, this capability is invaluable for modeling the complex interactions among genetic, clinical, and environmental factors influencing disease progression and treatment response (232). ML and DL algorithms have played a pivotal role in developing predictive models that inform risk stratification, prognosis, and therapeutic decisions(233).

One of AI’s key applications in RA involves enhancing diagnostic accuracy and disease classification. Traditional diagnosis relies on clinical assessment, laboratory tests, and imaging; however, AI models, such as ANNs and CNNs, have automated and improved these processes(234, 235). For instance, ANNs trained on demographic and serological data have achieved high accuracy in identifying RA patients. At the same time, CNNs applied to hand radiographs automate the detection of typical joint abnormalities, enabling earlier and more cost-effective diagnosis. These AI tools reduce human error and facilitate timely referral and intervention, which are critical to preventing disease progression(236–238). AI also shows promise in predicting the response to methotrexate (MTX), a cornerstone of RA treatment(239). One study used a penalized logistic regression model based on gene expression ratios before and after four weeks of MTX therapy, achieving an AUC of 0.78 in forecasting six-month treatment response(240). Comparisons of ML methods—including Lasso regression, random forest, and XGBoost—indicate that although advanced algorithms may provide slight improvements, traditional logistic regression remains competitive, with AUCs ranging from 0.77 to 0.78(241, 242).

Beyond predicting pharmacologic response, AI helps identify important prognostic biomarkers and risk factors in RA(209). For example, they are strong predictors of early response to tumor necrosis factor inhibitors (TNFi). These findings enhance patient stratification and facilitate clinicians in selecting the most optimal therapies, thereby reducing the reliance on trial-and-error approaches that have been historically common in RA treatment(243, 244).

AI is also poised to transform research in RA and drug development(245). By mining clinical trial data, biomedical literature, and real-world evidence, AI models can identify new therapeutic targets, optimize trial designs, and predict treatment outcomes, thereby accelerating the development of novel therapies. The multi-omics integration of genetic, proteomic, and clinical data has shown promise in predicting responses to biologics and Janus kinase (JAK) inhibitors. However, challenges remain regarding data heterogeneity and integration(246–248). Despite these advances, several challenges limit the full potential of AI in RA. The disease’s polygenic and multifactorial nature demands large and diverse datasets to develop robust, generalizable models. Variability in defining and measuring treatment responses, along with differing prediction timeframes across studies, complicates model comparison and clinical application. Moreover, issues of data quality, privacy, and ethics must be addressed to ensure the safe and effective deployment of AI in clinical practice(193, 249, 250).

### AI in type 1 diabetes

T1DM is a multifaceted autoimmune disease characterized by the infiltration of immune cells into pancreatic islets, resulting in the selective destruction of insulin-producing β-cells. This β-cell loss causes insulin deficiency and hyperglycemia, necessitating lifelong insulin replacement therapy(251). AI has significantly advanced diabetes care by enabling earlier and more precise diagnoses, optimizing treatment approaches, and supporting real-time disease management(252). AI and ML offer new avenues for early detection and prediction of diabetes, facilitating personalized interventions and improved outcomes. One critical application involves analyzing EHRs, which contain extensive patient data, including demographics, laboratory results, and medical history. AI/ML algorithms can detect patterns within this data that indicate diabetes or pre-diabetic conditions. Diabetes risk prediction models have been widely studied, with Schwarz et al. providing comprehensive analyses of their sensitivity and specificity(253, 254).

Accurate complication prediction is vital for targeted intervention to prevent or delay adverse outcomes(255). AI-powered predictive analytics hold great promise for enhancing management of chronic conditions by supporting patients in maintaining healthy lifestyles, adhering to medications, and effectively monitoring glycemic control(256, 257). Various AI algorithms, such as support vector machines, logistic regression, neural networks, and decision trees, are utilized in diabetes prediction(258).

**Fig. 5:**
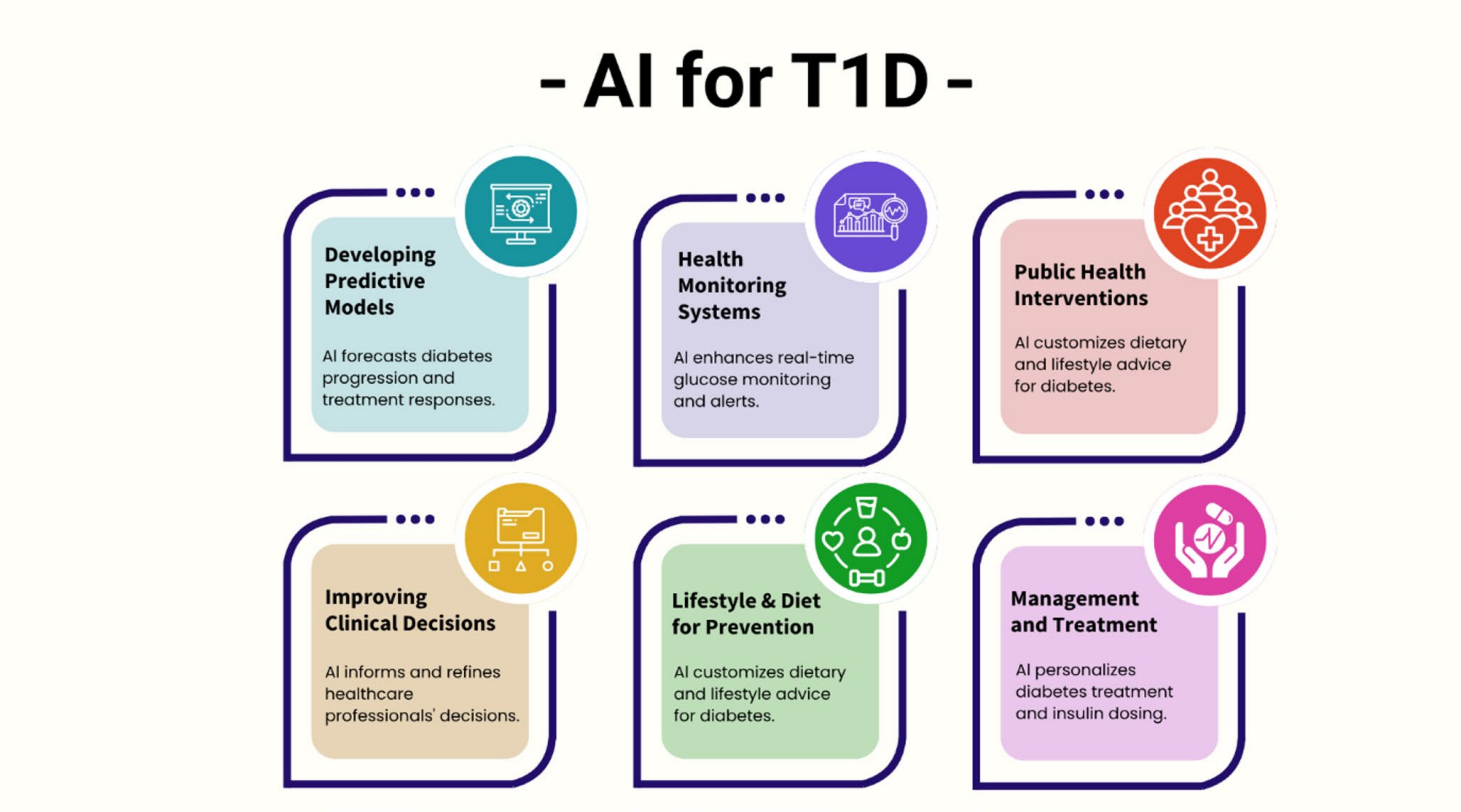
Schematic overview of AI applications in T1D. AI models are utilized across various domains, including predictive modeling of disease progression, real-time health monitoring, clinical decision-making, and personalized patient management. Additional roles include optimizing treatment strategies, supporting public health interventions, and tailoring lifestyle and dietary recommendations to enhance patient outcomes and enable precision diabetes care

These algorithms use diverse mathematical frameworks to recognize patterns and generate predictions. For example, Rajendra and Latifi applied logistic regression to the PIMA Indians Diabetes dataset (which primarily reflects T2DM risk rather than autoimmune T1DM) and a Vanderbilt dataset involving rural African Americans, achieving accuracies of 78% and 93%, respectively. Accordingly, predictive approaches based on PIMA (T2DM-oriented) should be distinguished from T1DM prediction models, which typically incorporate autoimmune/genetic markers (e.g., islet autoantibodies, HLA risk) and/or longitudinal glycemic trajectories. In addition, these accuracy values should be interpreted alongside sensitivity/specificity and AUC, and in the context of cohort characteristics (e.g., sample size, class balance, and population composition), which influence clinical utility and generalizability (259). Their findings highlight that factors beyond algorithm choice, such as thorough data preprocessing, removal of redundancies and missing values, normalization, cross-validation, feature selection, and ensemble methods, significantly impact model performance and efficiency(259, 260). Effective lifestyle management is a cornerstone of diabetes treatment, crucial for preventing complications and related health issues. Upon diagnosis, patients are encouraged to adopt healthier behaviors. Leveraging current technologies and extensive datasets, data-driven tools like Decision Support Systems (DSS) have been developed to empower patients and clinicians in diabetes management. These systems monitor diet, physical activity, medication adherence, glucose levels, and other relevant factors, thereby facilitating improved therapeutic outcomes(261). As illustrated in Fig. 5, AI technologies are revolutionizing T1DM care by enabling a comprehensive approach to disease prediction, real-time monitoring, and management. These systems support predictive modeling, guide individualized insulin therapy, inform lifestyle adjustments, and enhance clinical decision-making—ultimately driving more precise and responsive care tailored to each patient’s unique profile.

Drug discovery and development for T1DM increasingly rely on repurposing immunotherapies initially designed for other autoimmune diseases, transplantation, and cancer(262). These therapies target immunological pathways, including the depletion of T- and B-cells, inhibition of cytokines, and enhancement of regulatory T-cells. While some drugs are guided by T1DM-specific genetic risk loci (e.g., CTLA4, IL2RA), others act on broader mechanisms, such as polyclonal anti-thymocyte globulin and JAK inhibitors(263–265). Although these interventions have met primary clinical endpoints, they typically only transiently preserve endogenous C-peptide, underscoring the need for earlier, continuous, or combination therapies, as well as the development of agents with more durable effects. Advanced computational methods, particularly AI and ML, are being employed to identify promising drug candidates and predict off-target effects; however, few have progressed to clinical trials in T1DM to date(266, 267). Antigen-specific immunotherapies offer another promising strategy, with preliminary evidence indicating preservation of C-peptide levels in select T1DM subgroups(268, 269). The effectiveness of these therapies often depends on genetic factors such as HLA haplotypes, which influence immune responses. AI/ML tools are being integrated into quantitative trait locus analyses to model complex genetic effects on therapy response and predict peptides most likely presented by specific HLA molecules(270, 271). Unbiased immunopeptidome studies and advanced sequencing are identifying novel antigenic targets, while AI-driven databases and algorithms assist in discovering new immune receptor sequences and their antigen specificities. These advancements promise more precise immune tolerance induction strategies tailored to patient subpopulations(272–274).

Combination therapies are gaining interest for prolonging therapeutic efficacy in T1D. Clinical trials combining agents with different mechanisms, such as anti-IL-21 with liraglutide, have shown potential by addressing both inflammation and β-cell function. In this specific setting, AI/ML can support anti-IL-21 + liraglutide combination trials by integrating immunologic and metabolic biomarkers (e.g., inflammatory signatures together with C-peptide and glycemic trajectories) to identify responder subgroups, enable biomarker-guided monitoring, and evaluate treatment interactions/synergy(275–277). AI/ML-driven in silico models are increasingly used to predict drug interactions, toxicity, and synergistic effects, informing the design of safer and more effective combination treatments(278). These computational approaches are also applied to optimize β-cell regeneration and immune modulation, leveraging insights from cancer and transplantation research. Future studies will likely integrate both in vitro and in silico methods to guide therapy selection and dosing, minimizing adverse effects while maximizing benefit(279, 280). Precision medicine in T1DM advances through identifying responder subgroups and customizing therapies based on patient-specific features. While conventional statistics have identified immune signatures of responders’ post-treatment, predictive markers guiding therapy selection before initiation are needed(263, 281). Demographic (age, ethnicity), genetic (HLA, pharmacogenetic markers), and environmental factors contribute to variable drug responses(281–283). AI/ML models are being developed to integrate these factors, enabling the prediction of individual responses and the optimization of dosing regimens. The concept of “digital twins”—virtual patient models that incorporate genetic, immunological, and clinical data—holds promise for simulating therapeutic responses and refining precision medicine in T1D(284, 285).

Despite progress, challenges remain in clinically applying AI/ML to T1D therapy discovery. Ensuring model interpretability, generalizability, and actionability demands rigorous data management, interdisciplinary collaboration, and continuous expert engagement. The “black box” nature of many AI/ML approaches, DL, limits explainability, which is crucial for establishing clinical trust and adoption. Efforts to develop explainable AI (XAI) techniques aim to produce transparent outputs that clinicians and researchers can understand and validate. Bridging the gap between computational innovation and clinical relevance is essential to fully realize AI/ML’s potential in advancing T1D treatment(286–288).

### Algorithmic bias and equity in ai-driven immunotherapy

AI-enabled immunotherapy pipelines—spanning biomarker inference (e.g., PD-L1/TMB/MSI surrogates), response prediction, and immune-related adverse event (irAE) risk modeling—can amplify inequities when training and validation datasets do not reflect the populations intended for deployment(289). A central driver is the uneven composition and documentation of health datasets (“health data poverty”), which may result from structural barriers to research participation and persistent underrepresentation of certain demographic groups; consequently, model performance can degrade in precisely those settings where access to specialist oncology/immunology care is already limited, widening disparities in treatment selection and outcomes (290).

Bias can be introduced across the full AI lifecycle. Sampling bias occurs when genomic resources, clinical cohorts, trial-linked biobanks underrepresent specific ancestries/ethnicities, geographic regions, socioeconomic strata, or comorbidity profiles, undermining generalizability. Measurement and label bias arise when reference standards (“ground truth”) are inconsistently defined or measured across institutions—for example, variability in PD-L1 staining/scoring practices or sequencing/variant-calling pipelines—leading models to learn site- or subgroup-specific artifacts rather than biology. In parallel, dataset (distribution) shift across scanners, staining protocols, and clinical workflows can produce subgroup-specific degradation even when overall headline performance appears acceptable(290–292). In immuno-oncology, these concerns are particularly consequential because model outputs can influence high-stakes decisions such as ICI initiation, combination selection, and toxicity surveillance. When diversity in training/validation cohorts is limited or demographic reporting is incomplete, fairness and transportability become difficult to establish, increasing the risk of uneven benefit and harm across patient subgroups(289, 290). Equity risks also extend upstream: AI-enabled drug discovery and development workflows (including candidate prioritization and trial enrichment) can inherit biases from incomplete or unbalanced real-world and experimental datasets, which may distort discovery signals and limit downstream clinical applicability(290, 293). Mitigation therefore requires treating equity as a core performance dimension. Practical steps include documenting dataset composition and missingness, reporting stratified performance (e.g., calibration and AUC/sensitivity/specificity across relevant demographic and clinical subgroups), conducting external validation across institutions and workflows, and implementing post-deployment monitoring to detect drift and emergent subgroup harm. While fairness-aware learning and reweighting can help, they cannot substitute for representative data and consistent reference standards; governance and transparency are essential for equitable clinical translation(290, 292).

## Future Perspective

The integration of AI into immunotherapy marks a transformative shift in precision medicine for both cancer and autoimmune diseases(16). Looking ahead, several emerging directions are poised to reshape AI-driven Immunotherapeutics. In the near term, advancing explainable and interpretable AI will be essential for translating predictive models into clinically actionable tools. By making algorithmic outputs transparent and interpretable, AI will foster greater trust among clinicians, accelerate regulatory approval, and facilitate real-time decision-making in diverse clinical settings(39, 294).

However, routine clinical adoption remains limited not by model capability alone, but by translation barriers, most notably insufficient external validation across institutions and scanners/platforms, vulnerability to dataset shift, limited prospective and impact-focused clinical trials, and unresolved regulatory and clinical-evaluation requirements for AI deployed as software-as-a-medical-device (SaMD). These include a clear definition of intended use, clinically appropriate reference standards (“ground truth”), robust strategies for model updating and post-deployment performance monitoring, and ongoing surveillance for drift and safety(295, 296).

In parallel, immunotherapy relies on diverse datasets, including multi-omics, high-resolution imaging, and longitudinal clinical records, which must be integrated and harmonized across platforms and institutions(52). The rise of federated learning and privacy-preserving computation offers promising solutions, enabling collaborative model training without compromising patient privacy(297).

Yet, even with federated approaches, clinical-grade deployment requires standardized pre-analytic and analytic pipelines (e.g., acquisition/scanning protocols, sequencing batch correction, and complete metadata capture), rigorous quality control, and interoperable data governance to ensure that “real-world” inputs remain reliable enough for decision support (298, 299).

Over the longer term, digital twin technologies and in silico clinical trials are emerging as next-generation tools for therapy simulation(300). These patient-specific virtual models can dynamically predict disease trajectories and treatment responses, enabling preemptive optimization of dosing, safety, and efficacy before real-world administration(301). For example, in autoimmune disease, digital-twin (‘virtual patient’) models have been proposed for type 1 diabetes to simulate patient-specific glucose–insulin dynamics and immune–metabolic trajectories, supporting individualized insulin optimization and the in silico evaluation of immunomodulatory strategies; however, prospective clinical validation remains necessary before routine use(285).

This paradigm holds particular promise in reducing clinical trial costs and timelines(302), but its clinical translation will depend on demonstrating reproducible benefit against clinically meaningful endpoints in prospective evaluations, and meeting expectations for transparent verification/validation and safety monitoring (e.g., clearly defined intended use and reference standards, reporting algorithm versioning and input-data handling, systematic error analysis, and post-deployment surveillance for performance drift)(303). Additionally, the convergence of AI with next-generation biomedical technologies, such as single-cell transcriptomics, spatial omics, and 3D imaging, will uncover new layers of immune complexity. These integrative approaches are expected to reveal novel biomarkers, resistance mechanisms, and combinatorial therapeutic strategies, thereby expanding the therapeutic arsenal available to clinicians(304).

Ethical, legal, and societal considerations must evolve in harmony with technical advancements. Addressing algorithmic bias, ensuring data representativeness across populations, and developing transparent regulatory pathways will be essential for equitable AI deployment. Multidisciplinary collaboration among clinicians, data scientists, policymakers, and patient communities will be critical to ensuring responsible and inclusive innovation. In summary, the future of AI in immunotherapy will be defined not only by methodological advances, but also by robust validation, regulatory readiness, and implementation science that enable safe, effective, and scalable deployment across real-world clinical systems.

## Conclusion

AI is catalyzing a paradigm shift in immunotherapy, transforming the diagnosis, treatment, and monitoring of cancer and autoimmune diseases(16). By leveraging advanced ML and DL algorithms, AI enables the integration of complex biomedical data, including multi-omics, imaging, and real-world clinical records, into cohesive, clinically actionable insights(305). This data-driven transformation is propelling immunotherapy toward unprecedented levels of personalized treatment and efficacy(119).

AI has accelerated the identification of predictive biomarkers, including PD-L1, TMB, MSI, and neoantigen profiles in oncology(16, 33). These advances have improved patient stratification and enabled more precise deployment of ICIs(306). AI has also enhanced the design and monitoring of CAR-T cell therapies and antibody-based biopharmaceuticals by simulating molecular interactions, predicting toxicity, and optimizing dosing strategies, thereby shortening development timelines and improving safety profiles(65). In the realm of autoimmune disease, AI has deepened our understanding of disease heterogeneity, facilitated early diagnosis, and enabled personalized treatment(166). Through multi-omic analysis, wearable data integration, and EHR mining, AI supports longitudinal disease monitoring and drug repurposing in conditions such as RA, SLE, MS, and T1DM(21). These capabilities not only improve treatment outcomes but also open avenues for preventive and predictive care.

Despite these advancements, critical barriers remain. Data quality limitations, such as noise in single-cell RNA sequencing, batch effects in imaging, and inconsistencies in digital pathology staining, can compromise model performance. Inadequate quality control and dataset shifts across platforms exacerbate these issues, making model validation across institutions challenging. Equally important are interoperability challenges, insufficient standardization, and lack of regulatory frameworks for AI models, which remain significant hurdles to clinical adoption. Additionally, ethical considerations—including patient privacy, informed consent, accountability, and equitable access—must be embedded in the development and deployment of AI systems to ensure responsible translation into practice.

In conclusion, AI is a transformative force in immunotherapy, enhancing diagnostic accuracy, optimizing therapeutic efficacy, and advancing precision medicine. However, to fully realize its potential in clinical practice, continued investment in data infrastructure, model validation, and interdisciplinary collaboration will be essential. Ultimately, the goal is not just smarter algorithms—but more equitable, accessible, and human-centered care for all patients, everywhere.

## Data Availability

No datasets were generated or analysed during the current study.

## References

[1] Varadé J, Magadán S, González-Fernández Á. Human immunology and immunotherapy: main achievements and challenges. Cell Mol Immunol. 2021;18(4):805–28. 32879472 10.1038/s41423-020-00530-6 PMC7463107

[2] Han S, Shuen WH, Wang W-W, Nazim E, Toh HC. Tailoring precision immunotherapy: coming to a clinic soon? ESMO Open. 2020;5:e000631. 33558033 10.1136/esmoopen-2019-000631 PMC7046383

[3] Fitzmaurice C, Dicker D, Pain A, Hamavid H, Moradi-Lakeh M, MacIntyre MF et al. The global burden of cancer 2013. JAMA Oncol.1(4):505–27. 10.1001/jamaoncol.2015.0735 PMC4500822 26181261

[4] Balakrishna A, Nahrwold D, Hughes C. Intensive Care of Cancer Patients. Anesthesia for Oncological Surgery. Cham: Springer International Publishing. pp. 457–70.

[5] Boussios S, Pentheroudakis G, Katsanos K, Pavlidis N. Systemic treatment-induced gastrointestinal toxicity: incidence, clinical presentation, and management. Annals Gastroenterol.25(2):106&#8211. PMC3959393 24713845

[6] Xie J, Zheng Z, Tuo L, Deng X, Tang H, Peng C, et al. Recent advances in exosome-based immunotherapy applied to cancer. Front Immunol. 2023;14:1296857. 38022585 10.3389/fimmu.2023.1296857 PMC10662326

[7] Xu Z, Zeng S, Gong Z, Yan Y. Exosome-based immunotherapy: a promising approach for cancer treatment. Mol Cancer.19:1–16. 10.1186/s12943-020-01278-3 PMC7661275 33183286

[8] Ye Y, Zhang Y, Yang N, Gao Q, Ding X, Kuang X, et al. Profiling of immune features to predict immunotherapy efficacy. The Innovation. 2022. 10.1016/j.xinn.2021.100194. 34977836 10.1016/j.xinn.2021.100194 PMC8688727

[9] Nishino M, Ramaiya NH, Hatabu H, Hodi FS. Monitoring immune-checkpoint blockade: response evaluation and biomarker development. Nat Rev Clin Oncol. 2017;14(11):655–68. 28653677 10.1038/nrclinonc.2017.88 PMC5650537

[10] Li T, Li Y, Zhu X, He Y, Wu Y, Ying T, et al. Artificial intelligence in cancer immunotherapy: Applications in neoantigen recognition, antibody design and immunotherapy response prediction. Semin Cancer Biol. 2023;91:50–69. 36870459 10.1016/j.semcancer.2023.02.007

[11] Xie J, Luo X, Deng X, Tang Y, Tian W, Cheng H, et al. Advances in Artificial Intelligence to predict cancer immunotherapy efficacy. Front Immunol. 2023;13:1076883. 36685496 10.3389/fimmu.2022.1076883 PMC9845588

[12] Weber JK, Morrone JA, Kang SG, Zhang L, Lang L, Chowell D, et al. Unsupervised and supervised AI on molecular dynamics simulations reveal complex characteristics of HLA-A2-peptide immunogenicity. Brief Bioinform. 2024;25(1):504. 10.1093/bib/bbad504 PMC10793977 38233090

[13] Yang Y, Zhao Y, Liu X, Huang J. Artificial intelligence for the prediction of response to cancer immunotherapy. Seminars in Cancer Biology. 87: Academic Press. pp. 137–47. 10.1016/j.semcancer.2022.11.008 36372326

[14] D’Orsi L, Capasso B, Lamacchia G, Pizzichini P, Ferranti S, Liverani A, et al. Recent advances in artificial intelligence to improve immunotherapy and the use of digital twins to identify prognosis of patients with solid tumors. Int J Mol Sci. 2024;25(21):11588. 39519142 10.3390/ijms252111588 PMC11546512

[15] Costa Simões JM. A Machine Learning Approach for Predicting Microsatellite Instability using RNAseq.

[16] Olawade DB, Clement David-Olawade A, Adereni T, Egbon E, Teke J, Boussios S. Integrating AI into Cancer Immunotherapy—A. Narrative Rev Curr Appl Future Dir Dis.13(1):24&#8211. 10.3390/diseases13010024 PMC11764268 39851488

[17] Afzal M, Sah AK, Agarwal S, Tanzeel A, Elshaikh RH, Alobeidli FA, et al. Advancements in the treatment of autoimmune diseases: integrating artificial intelligence for personalized medicine. Trends in Immunotherapy. 2024. 10.24294/ti8970.

[18] Davidson A, Diamond B. Autoimmune diseases. N Engl J Med. 2001;345(5):340–50. 11484692 10.1056/NEJM200108023450506

[19] Ngo ST, Steyn FJ, McCombe PA. Gender differences in autoimmune disease. Front Neuroendocrinol. 2014;35(3):347–69. 24793874 10.1016/j.yfrne.2014.04.004

[20] Wang L, Khunsriraksakul C, Markus H, Chen D, Zhang F, Chen F, et al. Integrating single-cell expression quantitative trait loci summary statistics to understand complex trait risk genes. Nature communications. 2024;15(1):4260. 38769300 10.1038/s41467-024-48143-1 PMC11519974

[21] Stafford IS, Kellermann M, Mossotto E, Beattie RM, MacArthur BD, Ennis S. A systematic review of the applications of artificial intelligence and machine learning in autoimmune diseases. NPJ Digit Med. 2020;3(1):30. 32195365 10.1038/s41746-020-0229-3 PMC7062883

[22] Nwosu BU, Maranda L, Cullen K, Greenman L, Fleshman J, McShea N, et al. A randomized, double-blind, placebo-controlled trial of adjunctive metformin therapy in overweight/obese youth with type 1 diabetes. PLoS One. 2015;10(9):e0137525. 26367281 10.1371/journal.pone.0137525 PMC4569440

[23] Lund SS, Tarnow L, Astrup AS, Hovind P, Jacobsen PK, Alibegovic AC, et al. Effect of adjunct metformin treatment in patients with type-1 diabetes and persistent inadequate glycaemic control. A randomized study. PLoS One. 2008;3(10):e3363. 18852875 10.1371/journal.pone.0003363 PMC2566605

[24] Rider NL, Srinivasan R, Khoury P. Artificial intelligence and the hunt for immunological disorders. Curr Opin Allergy Clin Immunol. 2020;20(6):565–73. 33002894 10.1097/ACI.0000000000000691 PMC7908683

[25] Saxena S. OnionMHC: a deep learning model for peptide-HLA-A* 02: 01 binding predictions.

[26] Peng X, Yang D, Zhou Y, Peng S. TlcMHCpan: A Novel Deep Learning Model for Enhanced Pan-Specific Prediction of Peptide-HLA Binding: IEEE Access.

[27] Liu Z, Cui Y, Xiong Z, Nasiri A, Zhang A, Hu J. DeepSeqPan, a novel deep convolutional neural network model for pan-specific class I HLA-peptide binding affinity prediction. Sci Rep. 2019;9(1):794. 30692623 10.1038/s41598-018-37214-1 PMC6349913

[28] Su Z, Wu Y, Cao K, Du J, Cao L, Wu Z, et al. Apex-phla: a novel method for accurate prediction of the binding between exogenous short peptides and HLA class I molecules. Methods. 2024;228:38–47. 38772499 10.1016/j.ymeth.2024.05.013

[29] Bottomly D, McWeeney S. Just how transformative will AI/ML be for immuno-oncology? J Immunother Cancer. 2024;12(3):7841. 10.1136/jitc-2023-007841 PMC10966790 38531545

[30] Afkham SA, Mirdehghan S, Utopia. Can a New Era of AI Predict Immunotherapy Efficacy? Handbook of Cancer and Immunology. Cham: Springer Nature Switzerland; 2070. pp. 1–17.

[31] Dercle L, McGale J, Sun S, Marabelle A, Yeh R, Deutsch E, et al. Artificial intelligence and radiomics: fundamentals, applications, and challenges in immunotherapy. J Immunother Cancer. 2022;10(9):5292. 10.1136/jitc-2022-005292 PMC9528623 36180071

[32] Kang CY, Duarte SE, Kim HS, Kim E, Park J, Lee AD et al. Artificial Intelligence-based Radiomics in the Era of Immuno-oncology. Oncologist.27(6):471–83. 10.1093/oncolo/oyac036 PMC9177100 35348765

[33] Prelaj A, Miskovic V, Zanitti M, Trovo F, Genova C, Viscardi G, et al. Artificial intelligence for predictive biomarker discovery in immuno-oncology: a systematic review. Ann Oncol. 2024;35(1):29–65. 37879443 10.1016/j.annonc.2023.10.125

[34] Sun R, Henry T, Laville A, Carré A, Hamaoui A, Bockel S et al. Imaging approaches and radiomics: toward a new era of ultraprecision radioimmunotherapy? J Immunother Cancer.10(7):4848&#8211. 10.1136/jitc-2022-004848 PMC9260846 35793875

[35] Sako C, Duan C, Maresca K, Kent S, Schmidt TG, Aerts H, et al. Real-World and Clinical Trial Validation of a Deep Learning Radiomic Biomarker for PD-(L)1 Immune Checkpoint Inhibitor Response in Advanced Non-Small Cell Lung Cancer. JCO Clin Cancer Inf. 2024;8:e2400133. 10.1200/CCI.24.00133 PMC11658027 39671539

[36] Hill DLG. AI in imaging: the regulatory landscape. Br J Radiol. 2024;97(1155):483–91. 38366148 10.1093/bjr/tqae002 PMC11027239

[37] El Naqa I, Karolak A, Luo Y, Folio L, Tarhini AA, Rollison D, et al. Translation of AI into oncology clinical practice. Oncogene. 2023;42(42):3089–97. 37684407 10.1038/s41388-023-02826-z PMC12516697

[38] Li T, Li Y, Zhu X, He Y, Wu Y, Ying T et al. Artificial intelligence in cancer immunotherapy: Applications in neoantigen recognition, antibody design, and immunotherapy response prediction. Seminars in Cancer Biology. 91: Academic Press. pp. 50–69. 10.1016/j.semcancer.2023.02.007 36870459

[39] Yoo SK, Fitzgerald CW, Cho BA, Fitzgerald BG, Han C, Koh ES et al. Prediction of checkpoint inhibitor immunotherapy efficacy for cancer using routine blood tests and clinical data. Nature Medicine.1–12. 10.1038/s41591-024-03398-5 PMC11922749 39762425

[40] Rehmani MU, Aggarwal S, Prasad CP, Singh M. Dissecting the tumor microenvironment (TME) to decipher new immunotherapy targets by using artificial intelligence. Asian Pac J Cancer Care. 2024;9(4):793–9.

[41] Xu Z, Wang X, Zeng S, Ren X, Yan Y, Gong Z. Applying artificial intelligence for cancer immunotherapy. Acta Pharm Sin B. 2021;11(11):3393–405. 34900525 10.1016/j.apsb.2021.02.007 PMC8642413

[42] Zhang ZHE, Wei X. Artificial intelligence-assisted selection and efficacy prediction of antineoplastic strategies for precision cancer therapy. Seminars in Cancer Biology. 90: Academic Press. pp. 57–72. 10.1016/j.semcancer.2023.02.005 36796530

[43] Weerarathna IN, Kamble AR, Luharia A. Artificial intelligence applications for biomedical cancer research: a review. Cureus.15(11). 10.7759/cureus.48307 PMC10697339 38058345

[44] Elkhader J, Elemento O. Artificial intelligence in oncology: From bench to clinic. Seminars in Cancer Biology. 84: Academic Press. pp. 113–28. 10.1016/j.semcancer.2021.04.013 33915289

[45] Pardoll DM. The blockade of immune checkpoints in cancer immunotherapy. Nat Rev Cancer. 2012;12(4):252–64. 22437870 10.1038/nrc3239 PMC4856023

[46] Zou W, Chen L. Inhibitory B7-family molecules in the tumour microenvironment. Nat Rev Immunol. 2008;8(6):467–77. 18500231 10.1038/nri2326

[47] Ribas A, Wolchok JD. Cancer immunotherapy using checkpoint blockade. Science. 2018;359(6382):1350–5. 29567705 10.1126/science.aar4060 PMC7391259

[48] Addala V, Newell F, Pearson JV, Redwood A, Robinson BW, Creaney J, et al. Computational immunogenomic approaches to predict response to cancer immunotherapies. Nat Rev Clin Oncol. 2024;21(1):28–46. 37907723 10.1038/s41571-023-00830-6

[49] Alsaab HO, Sau S, Alzhrani R, Tatiparti K, Bhise K, Kashaw SK, et al. PD-1 and PD-L1 checkpoint signaling inhibition for cancer immunotherapy: mechanism, combinations, and clinical outcome. Front Pharmacol. 2017;8:561. 28878676 10.3389/fphar.2017.00561 PMC5572324

[50] Shamai G, Livne A, Polónia A, Sabo E, Cretu A, Bar-Sela G, et al. Deep learning-based image analysis predicts PD-L1 status from H&E-stained histopathology images in breast cancer. Nat Commun. 2022;13(1):6753. 36347854 10.1038/s41467-022-34275-9 PMC9643479

[51] Choi S, Cho SI, Ma M, Park S, Pereira S, Aum BJ et al. Artificial intelligence–powered programmed death ligand one analyser reduces interobserver variation in tumour proportion score for non–small cell lung cancer with better prediction of immunotherapy response. Eur J Cancer.170:17–26. 10.1016/j.ejca.2022.04.011 35576849

[52] Vanguri RS, Luo J, Aukerman AT, Egger JV, Fong CJ, Horvat N, et al. Multimodal integration of radiology, pathology and genomics for prediction of response to PD-(L) 1 blockade in patients with non-small cell lung cancer. Nat Cancer. 2022;3(10):1151–64. 36038778 10.1038/s43018-022-00416-8 PMC9586871

[53] Cheong JH, Wang SC, Park S, Porembka MR, Christie AL, Kim H, et al. Development and validation of a prognostic and predictive 32-gene signature for gastric cancer. Nat Commun. 2022;13(1):774. 35140202 10.1038/s41467-022-28437-y PMC8828873

[54] Gong X, Karchin R. Pan-cancer HLA gene-mediated tumor immunogenicity and immune evasion. Mol Cancer Res. 2022;20(8):1272–83. 35533264 10.1158/1541-7786.MCR-21-0886 PMC9357147

[55] Wang L, Zhang H, Pan C, Yi J, Cui X, Li N, et al. Predicting durable responses to immune checkpoint inhibitors in non-small-cell lung cancer using a multi-feature model. Front Immunol. 2022;13:829634. 35529874 10.3389/fimmu.2022.829634 PMC9072668

[56] Pan X, Zhang C, Wang J, Wang P, Gao Y, Shang S, et al. Epigenome signature as an immunophenotype indicator prompts durable clinical immunotherapy benefits in lung adenocarcinoma. Brief Bioinform. 2022;23(1):481. 10.1093/bib/bbab481 34864866

[57] Dercle L, Zhao B, Gönen M, Moskowitz CS, Firas A, Beylergil V, et al. Early readout on overall survival of patients with melanoma treated with immunotherapy using a novel imaging analysis. JAMA Oncol. 2022;8(3):385–92. 35050320 10.1001/jamaoncol.2021.6818 PMC8778619

[58] Paidi SK, Rodriguez Troncoso J, Raj P, Monterroso Diaz P, Ivers JD, Lee DE, et al. Raman spectroscopy and machine learning reveal early tumor microenvironmental changes induced by immunotherapy. Cancer Res. 2021;81(22):5745–55. 34645610 10.1158/0008-5472.CAN-21-1438 PMC8841097

[59] Tang T, Huang X, Zhang G, Hong Z, Bai X, Liang T. Advantages of targeting the tumor immune microenvironment over blocking immune checkpoints in cancer immunotherapy. Signal Transduct Target Ther. 2021;6(1):72. 33608497 10.1038/s41392-020-00449-4 PMC7896069

[60] Park S, Ock CY, Kim H, Pereira S, Park S, Ma M, et al. Artificial intelligence–powered spatial analysis of tumor-infiltrating lymphocytes as a complementary biomarker for immune checkpoint inhibition in non-small-cell lung cancer. J Clin Oncol. 2022;40(17):1916–28. 35271299 10.1200/JCO.21.02010 PMC9177249

[61] Zhang Z, Chen L, Chen H, Zhao J, Li K, Sun J, et al. Pan-cancer landscape of T-cell exhaustion heterogeneity within the tumor microenvironment revealed a progressive roadmap of hierarchical dysfunction associated with prognosis and therapeutic efficacy. EBioMedicine. 2022. 10.1016/j.ebiom.2022.104207. 35961204 10.1016/j.ebiom.2022.104207 PMC9382263

[62] Mo CK, Liu J, Chen S, Storrs E, Costa ALN, Houston A et al. Tumour evolution and microenvironment interactions in 2D and 3D space. Nature.634(8036):1178–86. 10.1038/s41586-024-08087-4 PMC11525187 39478210

[63] Özen M, Gündüz M, Haider H. Chimeric Antigen Receptor (CAR) T-Cells as a Therapeutic Modality. In: Haider H, editor Stem Cells: From Hype to. 8. Hope, WSP Company, Singapore, Chapter. p. 211–36.

[64] Luciani F, Safavi A, Guruprasad P, Chen L, Ruella M. Advancing CAR T-cell Therapies with Artificial Intelligence: Opportunities and Challenges. 10.1158/2643-3230.BCD-23-0240 PMC12050963 40152695

[65] Shahzadi M, Rafique H, Waheed A, Naz H, Waheed A, Zokirova FR, et al. Artificial intelligence for chimeric antigen receptor-based therapies: a comprehensive review of current applications and future perspectives. Ther Adv Vaccines Immunother. 2024;12:25151355241305856. 39691280 10.1177/25151355241305856 PMC11650588

[66] Jumper J, Evans R, Pritzel A, Green T, Figurnov M, Ronneberger O, et al. Highly accurate protein structure prediction with AlphaFold. Nature. 2021;596(7873):583–9. 34265844 10.1038/s41586-021-03819-2 PMC8371605

[67] Anurogo D, Luthfiana D, Anripa N, Fauziah AI, Soleha M, Rahmah L, et al. The art of Bioimmunogenomics (BIGs) 5.0 in CAR-T cell therapy for lymphoma management. Adv Pharm Bull. 2024;14(2):314. 39206402 10.34172/apb.2024.034 PMC11347730

[68] Barboy O, Katzenelenbogen Y, Shalita R, Amit I. In synergy: optimizing CAR T development and personalizing patient care using single-cell technologies. Cancer Discov. 2023;13(7):1546–55. 37219074 10.1158/2159-8290.CD-23-0010

[69] Kirouac DC, Zmurchok C, Deyati A, Sicherman J, Bond C, Zandstra PW. Deconvolution of clinical variance in CAR-T cell pharmacology and response. Nat Biotechnol. 2023;41(11):1606–17. 36849828 10.1038/s41587-023-01687-x PMC10635825

[70] Daher M, Rezvani K. Outlook for new CAR-based therapies with a focus on CAR NK cells: what lies beyond CAR-engineered T cells in the race against cancer. Cancer Discov. 2021;11(1):45–58. 33277313 10.1158/2159-8290.CD-20-0556 PMC8137521

[71] Daei Sorkhabi A, Mohamed Khosroshahi L, Sarkesh A, Mardi A, Aghebati-Maleki A, Aghebati-Maleki L, et al. The current landscape of CAR T-cell therapy for solid tumors: mechanisms, research progress, challenges, and counterstrategies. Front Immunol. 2023;14:1113882. 37020537 10.3389/fimmu.2023.1113882 PMC10067596

[72] Good Z, Spiegel JY, Sahaf B, Malipatlolla MB, Ehlinger ZJ, Kurra S, et al. Post-infusion CAR TReg cells identify patients resistant to CD19-CAR therapy. Nat Med. 2022;28(9):1860–71. 36097223 10.1038/s41591-022-01960-7 PMC10917089

[73] Yang C, Nguyen J, Yen Y. Complete spectrum of adverse events associated with chimeric antigen receptor (CAR)-T cell therapies. J Biomed Sci. 2023;30(1):89. 37864230 10.1186/s12929-023-00982-8 PMC10590030

[74] Wei L, El Naqa I. Artificial intelligence for response evaluation with PET/CT. Semin Nucl Med. 2021;51(2):157–69. 33509372 10.1053/j.semnuclmed.2020.10.003 PMC8099153

[75] Boretti A. The transformative potential of AI-driven CRISPR-Cas9 genome editing to enhance CAR T-cell therapy. Comput Biol Med. 2024;182:109137. 39260044 10.1016/j.compbiomed.2024.109137

[76] Zeng J, Lin Z, Zhang X, Zheng T, Xu H, Liu T. Leveraging artificial intelligence for neoantigen prediction. Cancer Res. 2025. 10.1158/0008-5472.CAN-24-2553. 40101113 10.1158/0008-5472.CAN-24-2553

[77] Carri I, Schwab E, Podaza E, Alvarez HMG, Mordoh J, Nielsen M et al. Beyond MHC binding: immunogenicity prediction tools to refine neoantigen selection in cancer patients. Explor Immunol.3(2):82–103.

[78] Nielsen M, Andreatta M, Peters B, Buus S. Immunoinformatics: predicting peptide–MHC binding. Annu Rev Biomed Data Sci. 2020;3(1):191–215. 37427310 10.1146/annurev-biodatasci-021920-100259 PMC10328453

[79] Weber D, Ibn-Salem J, Sorn P, Suchan M, Holtsträter C, Lahrmann U, et al. Accurate detection of tumor-specific gene fusions reveals strongly immunogenic personal neo-antigens. Nat Biotechnol. 2022;40(8):1276–84. 35379963 10.1038/s41587-022-01247-9 PMC7613288

[80] Pounraj S, Chen S, Ma L, Mazzieri R, Dolcetti R, Rehm BH. Targeting tumor heterogeneity with neoantigen-based cancer vaccines. Cancer Res. 2024;84(3):353–63. 38055891 10.1158/0008-5472.CAN-23-2042

[81] Huber F, Arnaud M, Stevenson BJ, Michaux J, Benedetti F, Thevenet J, et al. A comprehensive proteogenomic pipeline for neoantigen discovery to advance personalized cancer immunotherapy. Nat Biotechnol. 2025. 10.1038/s41587-024-02420-y. 39394480 10.1038/s41587-024-02420-y PMC12339364

[82] Baker TC. Improving detection and quantification of major histocompatibility complex (MHC)-presented immunopeptides for vaccine development.

[83] Greener JG, Kandathil SM, Moffat L, Jones DT. A guide to machine learning for biologists. Nat Rev Mol Cell Biol. 2022;23(1):40–55. 34518686 10.1038/s41580-021-00407-0

[84] Reynisson B, Alvarez B, Paul S, Peters B, Nielsen M. NetMHCpan-4.1 and NetMHCIIpan-4.0: improved predictions of MHC antigen presentation by concurrent motif deconvolution and integration of MS MHC eluted ligand data. Nucleic Acids Res.48(W1):449–54. 10.1093/nar/gkaa379 PMC7319546 32406916

[85] Nielsen M, Lundegaard C, Blicher T, Lamberth K, Harndahl M, Justesen S et al. NetMHCpan is a method for quantitative predictions of peptide binding to any HLA-A and- B locus protein of known sequence. PLoS ONE.2(8):796&#8211. 10.1371/journal.pone.0000796 PMC1949492 17726526

[86] O’Donnell TJ, Rubinsteyn A, Laserson U. MHCflurry 2.0: improved pan-allele prediction of MHC class I-presented peptides by incorporating antigen processing. Cell Syst.11(1):42–8. 10.1016/j.cels.2020.06.010 32711842

[87] Stražar M, Park J, Abelin JG, Taylor HB, Pedersen TK, Plichta DR et al. HLA-II immunopeptidome profiling and deep learning reveal antigenic features to inform antigen discovery. Immunity.56(7):1681–98. 10.1016/j.immuni.2023.05.009 PMC10519123 37301199

[88] Xu S, Wang X, Fei C. A highly effective system for predicting MHC-II epitopes with immunogenicity. Front Oncol. 2022;12:888556. 35785204 10.3389/fonc.2022.888556 PMC9246415

[89] Nilsson JB, Kaabinejadian S, Yari H, Kester MG, Balen P, Hildebrand WH et al. Accurate prediction of HLA class II antigen presentation across all loci using tailored data acquisition and refined machine learning. Sci Adv.9(47):6367&#8211. 10.1126/sciadv.adj6367 PMC10672173 38000035

[90] Reynisson B, Barra C, Kaabinejadian S, Hildebrand WH, Peters B, Nielsen M. Improved prediction of MHC II antigen presentation through integration and motif deconvolution of mass spectrometry MHC eluted ligand data. J Proteome Res. 2020;19(6):2304–15. 32308001 10.1021/acs.jproteome.9b00874

[91] Bradley P. Structure-based prediction of T cell receptor: peptide-MHC interactions. Elife. 2023;12:82813–. 10.7554/eLife.82813 PMC9859041 36661395

[92] Ghoreyshi ZS, George JT. Quantitative approaches for decoding the specificity of the human T cell repertoire. Front Immunol. 2023;14:1228873–. 37781387 10.3389/fimmu.2023.1228873 PMC10539903

[93] Bulashevska A, Nacsa Z, Lang F, Braun M, Machyna M, Diken M, et al. Artificial intelligence and neoantigens: paving the path for precision cancer immunotherapy. Front Immunol. 2024;15:1394003–. 38868767 10.3389/fimmu.2024.1394003 PMC11167095

[94] Kathuria KR, Chen B, Khodadoust MS, Olsson N, Davis MM, Elias JE, et al. Maria-I: a deep-learning approach for accurate prediction of MHC class I tumor neoantigen presentation. Blood. 2019;134:84–.

[95] Parkhurst MR, Robbins PF, Tran E, Prickett TD, Gartner JJ, Jia L, et al. Unique neoantigens arise from somatic mutations in patients with gastrointestinal cancers. Cancer Discov. 2019;9(8):1022–35. 31164343 10.1158/2159-8290.CD-18-1494 PMC7138461

[96] Powderly JD, Sullivan RJ, Gutierrez M, Khattak A, Thomas SS, Jimeno A et al. Phase 1/2 study of mRNA-4359 administered alone and in combination with immune checkpoint blockade in adult participants with advanced solid tumors.

[97] Xu Y. Deep neural networks for QSAR. Artificial Intelligence in Drug Design. New York, NY: Springer US. pp. 233–60.

[98] Bahrami Y, Bolideei M, Mohammadzadeh S, Gahrouei RB, Mohebbi E, Haider KH, et al. Applications of artificial intelligence and nanotechnology in vaccine development. Int J Pharm. 2025;684:126096. 40886810 10.1016/j.ijpharm.2025.126096

[99] Rojas LA, Sethna Z, Soares KC, Olcese C, Pang N, Patterson E, et al. Personalized RNA neoantigen vaccines stimulate T cells in pancreatic cancer. Nature. 2023;618(7963):144–50. 37165196 10.1038/s41586-023-06063-y PMC10171177

[100] Lin YJ, Zimmermann J, Schülke S. Novel adjuvants in allergen-specific immunotherapy: where do we stand? Front Immunol. 2024;15:1348305. 38464539 10.3389/fimmu.2024.1348305 PMC10920236

[101] Haider S, Barakat A, Ul-Haq Z. Discovery of potential chemical probe as inhibitors of CXCL12 using ligand-based virtual screening and molecular dynamic simulation. Molecules. 2020;25(20):4829. 33092204 10.3390/molecules25204829 PMC7594044

[102] Zhang WY, Zheng XL, Coghi PS, Chen JH, Dong BJ, Fan XX. Revolutionizing adjuvant development: harnessing AI for next-generation cancer vaccines. Front Immunol. 2024;15:1438030. 39206192 10.3389/fimmu.2024.1438030 PMC11349682

[103] Vandenberghe ME, Scott ML, Scorer PW, Söderberg M, Balcerzak D, Barker C. Relevance of deep learning to facilitate the diagnosis of HER2 status in breast cancer. Sci Rep. 2017;7(1):45938. 28378829 10.1038/srep45938 PMC5380996

[104] Palm C, Connolly CE, Masser R, Padberg Sgier B, Karamitopoulou E, Simon Q, et al. Determining HER2 status by artificial intelligence: an investigation of primary, metastatic, and HER2 low breast tumors. Diagnostics Basel. 2023;13(1):168. 36611460 10.3390/diagnostics13010168 PMC9818571

[105] Potočnik J, Foley S, Thomas E. Current and potential applications of artificial intelligence in medical imaging practice: a narrative review. J Med Imaging Radiat Sci. 2023;54(2):376–85. 37062603 10.1016/j.jmir.2023.03.033

[106] Geis JR, Brady AP, Wu CC, Spencer J, Ranschaert E, Jaremko JL, et al. Ethics of artificial intelligence in radiology: summary of the joint European and North American multisociety statement. Radiology. 2019;293(2):436–40. 31573399 10.1148/radiol.2019191586

[107] Gong J, Bao X, Wang T, Liu J, Peng W, Shi J, et al. A short-term follow-up CT-based radiomics approach to predict response to immunotherapy in advanced non-small-cell lung cancer. Oncoimmunology. 2022;11(1):2028962. 35096486 10.1080/2162402X.2022.2028962 PMC8794258

[108] He B, Dong D, She Y, Zhou C, Fang M, Zhu Y, et al. Predicting response to immunotherapy in advanced non-small-cell lung cancer using tumor mutational burden radiomic biomarker. J Immunother Cancer. 2020;8(2):550. 10.1136/jitc-2020-000550 PMC7342823 32636239

[109] Tosta TAA, Faria PR, Neves LA, Nascimento MZ. Color normalization of faded H&E-stained histological images using spectral matching. Comput Biol Med.111:103344&#8211. 10.1016/j.compbiomed.2019.103344 31279982

[110] Vijh S, Saraswat M, Kumar S. A new complete color normalization method for H&E-stained histopathological images. Appl Intell.51(11):7735–48.

[111] Sirinukunwattana K, Raza SEA, Tsang YW, Snead DR, Cree IA, Rajpoot NM. Locality sensitive deep learning for detection and classification of nuclei in routine colon cancer histology images. IEEE Trans Med Imaging. 2016;35(5):1196–206. 26863654 10.1109/TMI.2016.2525803

[112] Altini N, Brunetti A, Puro E, Taccogna MG, Saponaro C, Zito FA, et al. Ndg-cam: Nuclei detection in histopathology images with semantic segmentation networks and grad-cam. Bioengineering. 2022;9(9):475. 36135021 10.3390/bioengineering9090475 PMC9495364

[113] Chang L, Liu J, Zhu J, Guo S, Wang Y, Zhou Z et al. Advancing precision medicine: the transformative role of artificial intelligence in immunogenomics, radiomics, and pathomics for biomarker discovery and immunotherapy optimization. Cancer Biology Med.22(1):33–47. 10.20892/j.issn.2095-3941.2024.0376 PMC11795263 39749734

[114] Tao W, Sun Q, Xu B, Wang R. Towards the prediction of responses to cancer immunotherapy: a multi-omics review. Life. 2025;15(2):283. 40003691 10.3390/life15020283 PMC11856636

[115] Kong J, Ha D, Lee J, Kim I, Park M, Im SH, et al. Network-based machine learning approach to predict immunotherapy response in cancer patients. Nat Commun. 2022;13(1):3703. 35764641 10.1038/s41467-022-31535-6 PMC9240063

[116] Li S, Li W, Ma T, Fu S, Gao X, Qin N et al. Assessing the efficacy of immunotherapy in lung squamous carcinoma using an artificial intelligence neural network. Front Immunol.13:1024707&#8211. 10.3389/fimmu.2022.1024707 PMC9742243 36518765

[117] Kather JN, Pearson AT, Halama N, Jäger D, Krause J, Loosen SH, et al. Deep learning can predict microsatellite instability directly from histology in gastrointestinal cancer. Nat Med. 2019;25(7):1054–6. 31160815 10.1038/s41591-019-0462-y PMC7423299

[118] Tapper W, Carneiro G, Mikropoulos C, Thomas SA, Evans PM, Boussios S. The application of radiomics and AI to molecular imaging for prostate cancer. J Pers Med. 2024;14(3):287. 38541029 10.3390/jpm14030287 PMC10971024

[119] Ozaki Y, Broughton P, Abdollahi H, Valafar H, Blenda AV. Integrating omics data and AI for cancer diagnosis and prognosis. Cancers. 2024;16(13):2448. 39001510 10.3390/cancers16132448 PMC11240413

[120] Casak SJ, Marcus L, Fashoyin-Aje L, Mushti SL, Cheng J, Shen YL, et al. FDA approval summary: pembrolizumab for the first-line treatment of patients with MSI-H/dMMR advanced unresectable or metastatic colorectal carcinoma. Clin Cancer Res. 2021;27(17):4680–4. 33846198 10.1158/1078-0432.CCR-21-0557 PMC8416693

[121] Pembrolizumab for previously. treated, microsatellite instability–high/mismatch repair–deficient advanced colorectal cancer: final analysis of KEYNOTE-164. Eur J Cancer.186:185–95. 10.1016/j.ejca.2023.02.016 37141828

[122] Priestley P, Baber J, Lolkema MP, Steeghs N, Bruijn E, Shale C, et al. Pan-cancer whole-genome analyses of metastatic solid tumours. Nature. 2019;575(7781):210–6. 31645765 10.1038/s41586-019-1689-y PMC6872491

[123] Bao X, Zhang H, Wu W, Cheng S, Dai X, Zhu X et al. Analysis of the molecular nature associated with microsatellite status in colon cancer identifies clinical implications for immunotherapy—Journal for immunotherapy of cancer. p. 1437&#8211. 10.1136/jitc-2020-001437 PMC7542666 33028695

[124] Lu C, Zhang YC, Chen ZH, Zhou Q, Wu YL. Applications of circulating tumor DNA in immune checkpoint inhibition: emerging roles and future perspectives. Front Oncol. 2022;12:836891. 35359372 10.3389/fonc.2022.836891 PMC8963952

[125] Saillard C, Dubois R, Tchita O, Loiseau N, Garcia T, Adriansen A, et al. Validation of MSIntuit as an AI-based pre-screening tool for MSI detection from colorectal cancer histology slides. Nat Commun. 2023;14(1):6695. 37932267 10.1038/s41467-023-42453-6 PMC10628260

[126] Koelzer VH, Gisler A, Hanhart JC, Griss J, Wagner SN, Willi N et al. Digital image analysis enhances the precision of PD-L1 scoring in cutaneous melanoma. Histopathology.73(3):397–406. 10.1111/his.13528 29660160

[127] Schmitt AM, Larkin J, Patel SP. Dual immune checkpoint inhibition in melanoma and PD-L1 expression: the jury is still out. J Clin Oncol. 2025;43(2):122–4. 39374477 10.1200/JCO-24-01572

[128] Wu J, Liu C, Liu X, Sun W, Li L, Gao N, et al. Artificial intelligence-assisted system for precision diagnosis of PD-L1 expression in non-small cell lung cancer. Mod Pathol. 2022;35(3):403–11. 34518630 10.1038/s41379-021-00904-9

[129] Chan TA, Yarchoan M, Jaffee E, Swanton C, Quezada SA, Stenzinger A, et al. Development of tumor mutation burden as an immunotherapy biomarker: utility for the oncology clinic. Ann Oncol. 2019;30(1):44–56. 30395155 10.1093/annonc/mdy495 PMC6336005

[130] Moeckel C, Bakhl K, Georgakopoulos-Soares I, Zaravinos A. The efficacy of tumor mutation burden as a biomarker of response to immune checkpoint inhibitors. Int J Mol Sci. 2023;24(7):6710. 37047684 10.3390/ijms24076710 PMC10095310

[131] Jain MS, Massoud TF. Predicting tumour mutational burden from histopathological images using multiscale deep learning. Nat Mach Intell. 2020;2(6):356–62.

[132] Dewaker V, Morya VK, Kim YH, Park ST, Kim HS, Koh YH. Revolutionizing oncology: the role of Artificial Intelligence (AI) as an antibody design and optimization tool. Biomark Res.13(1):52&#8211. 10.1186/s40364-025-00764-4 PMC11954232 40155973

[133] Cheng J, Liang T, Xie XQ, Feng Z, Meng L. A new era of antibody discovery: an in-depth review of AI-driven approaches. Drug Discov Today. 2024. 10.1016/j.drudis.2024.103984. 38642702 10.1016/j.drudis.2024.103984

[134] Zhang DE, He T, Shi T, Huang K, Peng A. Trends in the research and development of peptide drug conjugates: Artificial Intelligence-aided design. Front Pharmacol. 2025;16:1553853. 40083376 10.3389/fphar.2025.1553853 PMC11903715

[135] Zhang Y, Luo M, Wu P, Wu S, Lee T-Y, Bai C. Application of computational biology and artificial intelligence in drug design. Int J Mol Sci. 2022;23(21):13568. 36362355 10.3390/ijms232113568 PMC9658956

[136] Georgiou G, Ippolito GC, Beausang J, Busse CE, Wardemann H, Quake SR. The promise and challenge of high-throughput sequencing of the antibody repertoire. Nat Biotechnol. 2014;32(2):158–68. 24441474 10.1038/nbt.2782 PMC4113560

[137] Liu G, Zeng H, Mueller J, Carter B, Wang Z, Schilz J, et al. Antibody complementarity-determining region design using high-capacity machine learning. Bioinformatics. 2020;36(7):2126–33. 31778140 10.1093/bioinformatics/btz895 PMC7141872

[138] Mason DM, Friedensohn S, Weber CR, Jordi C, Wagner B, Meng SM, et al. Optimization of therapeutic antibodies by predicting antigen specificity from antibody sequence via deep learning. Nat Biomed Eng. 2021;5(6):600–12. 33859386 10.1038/s41551-021-00699-9

[139] Makowski EK, Kinnunen PC, Huang J, Wu L, Smith MD, Wang T et al. Co-optimization of therapeutic antibody affinity and specificity using machine learning models that generalize to novel mutational space. Nat Commun.13(1):3788&#8211. 10.1038/s41467-022-31457-3 PMC9249733 35778381

[140] Saka K, Kakuzaki T, Metsugi S, Kashiwagi D, Yoshida K, Wada M et al. Antibody design using an LSTM-based deep generative model from the phage display library for affinity maturation. Sci Rep.11(1):5852&#8211. 10.1038/s41598-021-85274-7 PMC7955064 33712669

[141] Baek M, DiMaio F, Anishchenko I, Dauparas J, Ovchinnikov S, Lee GR, et al. Accurate prediction of protein structures and interactions using a three-track neural network. Science. 2021;373(6557):871–6. 34282049 10.1126/science.abj8754 PMC7612213

[142] Xu J. Distance-based protein folding powered by deep learning. Proc Natl Acad Sci U S A. 2019;116(34):16856–65. 31399549 10.1073/pnas.1821309116 PMC6708335

[143] He K, Zhang X, Ren S, Sun J, editors. Deep residual learning for image recognition. Proceedings of the IEEE conference on computer vision and pattern recognition.

[144] Ruffolo JA, Sulam J, Gray JJ. Antibody structure prediction using interpretable deep learning. Patterns. 2022. 10.1016/j.patter.2021.100406. 35199061 10.1016/j.patter.2021.100406 PMC8848015

[145] Raybould MI, Marks C, Krawczyk K, Taddese B, Nowak J, Lewis AP, et al. Five computational developability guidelines for therapeutic antibody profiling. Proc Natl Acad Sci U S A. 2019;116(10):4025–30. 30765520 10.1073/pnas.1810576116 PMC6410772

[146] Abanades B, Georges G, Bujotzek A, Deane CM. ABlooper: fast, accurate antibody CDR loop structure prediction with accuracy estimation. Bioinformatics. 2022;38(7):1877–80. 35099535 10.1093/bioinformatics/btac016 PMC8963302

[147] Satorras VG, Hoogeboom E, Welling M, editors. E (n) equivariant graph neural networks. International Conference on machine learning: PMLR.

[148] Cohen T, Halfon M, Schneidman-Duhovny D. NanoNet: rapid and accurate end-to-end nanobody modeling by deep learning. Front Immunol. 2022;13:958584. 36032123 10.3389/fimmu.2022.958584 PMC9411858

[149] Davila A, Xu Z, Li S, Rozewicki J, Wilamowski J, Kotelnikov S, et al. AbAdapt: an adaptive approach to predicting antibody–antigen complex structures from sequence. Bioinform Adv. 2022. 10.1093/bioadv/vbac015. 36699363 10.1093/bioadv/vbac015 PMC9710585

[150] Xu Z, Davila A, Wilamowski J, Teraguchi S, Standley DM. Improved antibody-specific epitope prediction using alphafold and abadapt. Chembiochem. 2022;23(18):202200303. 10.1002/cbic.202200303 PMC9543094 35893479

[151] Jarasch A, Koll H, Regula JT, Bader M, Papadimitriou A, Kettenberger H. Developability assessment during the selection of novel therapeutic antibodies. J Pharm Sci. 2015;104(6):1885–98. 25821140 10.1002/jps.24430

[152] Xu Y, Wang D, Mason B, Rossomando T, Li N, Liu D, et al. Structure, heterogeneity, and developability assessment of therapeutic antibodies. MAbs. 2019;11(2):239–64. 30543482 10.1080/19420862.2018.1553476 PMC6380400

[153] Wollacott AM, Xue C, Qin Q, Hua J, Bohnuud T, Viswanathan K, et al. Quantifying the nativeness of antibody sequences using long short-term memory networks. Protein Eng Des Sel. 2019;32(7):347–54. 31504835 10.1093/protein/gzz031 PMC7372931

[154] Prihoda D, Maamary J, Waight A, Juan V, Fayadat-Dilman L, Svozil D, et al. BioPhi: a platform for antibody design, humanization, and humanness evaluation based on natural antibody repertoires and deep learning. MAbs. 2022;14(1):2020203. 35133949 10.1080/19420862.2021.2020203 PMC8837241

[155] Lai PK. Deepscm: an efficient convolutional neural network surrogate model for the screening of therapeutic antibody viscosity. Comput Struct Biotechnol J. 2022;20:2143–52. 35832619 10.1016/j.csbj.2022.04.035 PMC9092385

[156] Agrawal NJ, Helk B, Kumar S, Mody N, Sathish HA, Samra HS, et al. Computational tool for the early screening of monoclonal antibodies for their viscosities. MAbs. 2016;8(1):43–8. 26399600 10.1080/19420862.2015.1099773 PMC4966561

[157] Wu H, Pfarr DS, Johnson S, Brewah YA, Woods RM, Patel NK, et al. Development of motavizumab, an ultra-potent antibody for the prevention of respiratory syncytial virus infection in the upper and lower respiratory tract. J Mol Biol. 2007;368(3):652–65. 17362988 10.1016/j.jmb.2007.02.024

[158] Kelly RL, Yu Y, Sun T, Caffry I, Lynaugh H, Brown M, et al. Target-independent variable region-mediated effects on antibody clearance can be FcRn-independent. MAbs. 2016;8(7):1269–75. 27610650 10.1080/19420862.2016.1208330 PMC5058615

[159] Grinshpun B, Thorsteinson N, Pereira JN, Rippmann F, Nannemann D, Sood VD, et al. Identifying biophysical assays and in silico properties that enrich for slow clearance in clinical-stage therapeutic antibodies. MAbs. 2021;13(1):1932230. 34116620 10.1080/19420862.2021.1932230 PMC8204999

[160] Kumar S, Kaushik D, Sharma SK. Autoimmune disorders: Types, symptoms, and risk factors. Artificial intelligence and autoimmune diseases: applications in the diagnosis, prognosis, and therapeutics. pp. 3–31.

[161] Mane DV, Deshmukh AN, Ambare RH, Solankar AA, Madane CS. AI in autoimmune diseases: transforming diagnosis and treatment. J Pharm Biol Sci. 2025;12(2):109–18.

[162] Laigle L, Chadli L, Moingeon P. Biomarker-driven development of new therapies for autoimmune diseases: current status and future promises. Expert Rev Clin Immunol. 2023;19(3):305–14. 36680799 10.1080/1744666X.2023.2172404

[163] Jorge A, Castro VM, Barnado A, Gainer V, Hong C, Cai T, et al. Identifying lupus patients in electronic health records: development and validation of machine learning algorithms and application of rule-based algorithms. Semin Arthritis Rheum. 2019;49(1):84–90. 30665626 10.1016/j.semarthrit.2019.01.002 PMC6609504

[164] Sorkhabi MA, Potapenko IO, Ilginis T, Alberti M, Cabrerizo J. Assessment of anterior uveitis through anterior-segment optical coherence tomography and artificial intelligence-based image analyses. Transl Vis Sci Technol. 2022;11(4):7. 35394486 10.1167/tvst.11.4.7 PMC8994203

[165] Haggag S, Khalifa F, Abdeltawab H, Elnakib A, Ghazal M, Mohamed MA, et al. An automated CAD system for accurate grading of uveitis using optical coherence tomography images. Sensors. 2021. 10.3390/s21165457. 34450898 10.3390/s21165457 PMC8401645

[166] Liu S, Liu Y, Li M, Shang S, Cao Y, Shen X, et al. Artificial intelligence in autoimmune diseases: a bibliometric exploration of the past two decades. Front Immunol. 2025;16:1525462. 40330462 10.3389/fimmu.2025.1525462 PMC12052778

[167] Raza K, Singh NK. A tour of unsupervised deep learning for medical image analysis. Curr Med Imaging Rev. 2021;17(9):1059–77. 10.2174/1573405617666210127154257 33504314

[168] Wang J, Wang S, Zhang Y. Deep learning on medical image analysis. CAAI Trans Intell Technol. 2025;10(1):1–35.

[169] Ahn JC, Connell A, Simonetto DA, Hughes C, Shah VH. Application of artificial intelligence for the diagnosis and treatment of liver diseases. Hepatology. 2021;73(6):2546–63. 33098140 10.1002/hep.31603

[170] Chen H, Sung JJ. Potentials of AI in medical image analysis in Gastroenterology and Hepatology. J Gastroenterol Hepatol. 2021;36(1):31–8. 33140875 10.1111/jgh.15327

[171] Zhuang H, Zhang J, Liao F. A systematic review on the application of deep learning in digestive system image processing. Visual Comput.39(6):2207–22. 10.1007/s00371-021-02322-z PMC8557108 34744231

[172] Christou CD, Tsoulfas G. Challenges and opportunities in the application of artificial intelligence in gastroenterology and hepatology. World J Gastroenterol. 2021;27(37):6191. 34712027 10.3748/wjg.v27.i37.6191 PMC8515803

[173] Wu H, Yin H, Chen H, Sun M, Liu X, Yu Y, et al. A deep learning, image based approach for automated diagnosis for inflammatory skin diseases. Ann Transl Med. 2020;8(9):581. 32566608 10.21037/atm.2020.04.39 PMC7290553

[174] Siddiqui MF, Mouna A, Nicolas G, Rahat SAA, Mitalipova A, Emmanuel N et al. Computational intelligence: a step forward in cancer biomarker discovery and therapeutic target prediction. Computational Intelligence in Oncology: Applications in Diagnosis, Prognosis and Therapeutics of Cancers. Singapore: Springer Singapore. pp. 233–50.

[175] Mishra A, Sharma S, Pandey SK. The Present State and Potential Applications of Artificial Intelligence in Cancer Diagnosis and Treatment. Recent Patents on Anti-Cancer Drug Discovery. 10.2174/0115748928361472250123105507 39902536

[176] Khalifa A, Obeid JS, Erno J, Rockey DC. The role of artificial intelligence in hepatology research and practice. Curr Opin Gastroenterol. 2023;39(3):175–80. 37144534 10.1097/MOG.0000000000000926

[177] Zhou XQ, Huang S, Shi XM, Liu S, Zhang W, Shi L, et al. Global trends in artificial intelligence applications in liver disease over seventeen years. World J Hepatol. 2025;17(3):101721. 40177211 10.4254/wjh.v17.i3.101721 PMC11959664

[178] Krittanawong C, Zhang H, Wang Z, Aydar M, Kitai T. Artificial intelligence in precision cardiovascular medicine. J Am Coll Cardiol. 2017;69(21):2657–64. 28545640 10.1016/j.jacc.2017.03.571

[179] Manlhiot C, Eynde J, Kutty S, Ross HJ. A primer on the present state and prospects for machine learning and artificial intelligence applications in cardiology. Can J Cardiol.38(2):169–84. 10.1016/j.cjca.2021.11.009 34838700

[180] Labkoff S, Oladimeji B, Kannry J, Solomonides A, Leftwich R, Koski E, et al. Toward a responsible future: recommendations for AI-enabled clinical decision support. J Am Med Inform Assoc. 2024;31(11):2730–9. 39325508 10.1093/jamia/ocae209 PMC11491642

[181] Karalis VD. The integration of artificial intelligence into clinical practice. Appl Biosci. 2024;3(1):14–44.

[182] Shaikh F, Dehmeshki J, Bisdas S, Roettger-Dupont D, Kubassova O, Aziz M, et al. Artificial intelligence-based clinical decision support systems using advanced medical imaging and radiomics. Curr Probl Diagn Radiol. 2021;50(2):262–7. 32591104 10.1067/j.cpradiol.2020.05.006

[183] Boeken T, Feydy J, Lecler A, Soyer P, Feydy A, Barat M, et al. Artificial intelligence in diagnostic and interventional radiology: where are we now? Diagn Interv Imaging. 2023;104(1):1–5. 36494290 10.1016/j.diii.2022.11.004

[184] Wong GLH, Yuen PC, Ma AJ, Chan AWH, Leung HHW, Wong VWS. Artificial intelligence in prediction of non-alcoholic fatty liver disease and fibrosis. J Gastroenterol Hepatol. 2021;36(3):543–50. 33709607 10.1111/jgh.15385

[185] Lu F, Meng Y, Song X, Li X, Liu Z, Gu C, et al. Artificial intelligence in liver diseases: recent advances. Adv Ther. 2024;41(3):967–90. 38286960 10.1007/s12325-024-02781-5

[186] Siddiqui MF, Alam A, Kalmatov R, Mouna A, Villela R, Mitalipova A et al. Leveraging healthcare system with nature-inspired computing techniques: an overview and future perspective. Nature-Inspired Intelligent Computing Techniques in Bioinformatics. pp. 19–42.

[187] Su TH, Wu CH, Kao JH. Artificial intelligence in precision medicine in hepatology. J Gastroenterol Hepatol. 2021;36(3):569–80. 33709606 10.1111/jgh.15415

[188] Schattenberg JM, Chalasani N, Alkhouri N. Artificial intelligence applications in hepatology. Clin Gastroenterol Hepatol. 2023;21(8):2015–25. 37088460 10.1016/j.cgh.2023.04.007

[189] Qazi S, Iqbal N, Raza K. Fuzzy logic-based hybrid models for clinical decision support systems in cancer. Computational Intelligence in Oncology: Applications in Diagnosis, Prognosis and Therapeutics of Cancers. Singapore: Springer Singapore. pp. 201–13.

[190] Le Berre C, Sandborn WJ, Aridhi S, Devignes MD, Fournier L, Smaïl-Tabbone M et al. Application of artificial intelligence to gastroenterology and hepatology. Gastroenterology.158(1):76–94. 10.1053/j.gastro.2019.08.058 31593701

[191] Calderaro J, Žigutytė L, Truhn D, Jaffe A, Kather JN. Artificial intelligence in liver cancer—new tools for research and patient management. Nat Rev Gastroenterol Hepatol. 2024;21(8):585–99. 38627537 10.1038/s41575-024-00919-y

[192] Rajkomar A, Hardt M, Howell MD, Corrado G, Chin MH. Ensuring fairness in machine learning to advance health equity. Ann Intern Med. 2018;169(12):866–72. 30508424 10.7326/M18-1990 PMC6594166

[193] Yang Y, Liu Y, Chen Y, Luo D, Xu K, Zhang L. Artificial intelligence for predicting treatment responses in autoimmune rheumatic diseases: advancements, challenges, and future perspectives. Front Immunol. 2024;15:1477130. 39502698 10.3389/fimmu.2024.1477130 PMC11534874

[194] Moingeon P. Artificial intelligence-driven drug development against autoimmune diseases. Trends Pharmacol Sci. 2023;44(7):411–24. 37268540 10.1016/j.tips.2023.04.005

[195] Cheng X, Meng X, Chen R, Song Z, Li S, Wei S, et al. The molecular subtypes of autoimmune diseases. Comput Struct Biotechnol J. 2024. 10.1016/j.csbj.2024.03.026. 38596313 10.1016/j.csbj.2024.03.026 PMC11001648

[196] Conte L, Caruso G, Philip AK, Cucci F, Nunzio G, Cascio D et al. Artificial Intelligence-Assisted Drug and Biomarker Discovery for Glioblastoma: A Scoping Review of the Literature. Cancers.17(4):571&#8211. 10.3390/cancers17040571 PMC11852502 40002166

[197] Jiang P, Sinha S, Aldape K, Hannenhalli S, Sahinalp C, Ruppin E. Big data in basic and translational cancer research. Nat Rev Cancer. 2022;22(11):625–39. 36064595 10.1038/s41568-022-00502-0 PMC9443637

[198] Biswas N, Chakrabarti S. Artificial intelligence (AI)-based systems biology approaches in the analysis of multi-omics data for cancer. Front Oncol.10:588221&#8211. 10.3389/fonc.2020.588221 PMC7591760 33154949

[199] Rehman AU, Li M, Wu B, Ali Y, Rasheed S, Shaheen S et al. Role of artificial intelligence in revolutionizing drug discovery: Fundamental Research. 10.1016/j.fmre.2024.04.021 PMC12167903 40528990

[200] Saini JPS, Thakur A, Yadav D. AI-driven Innovations in Pharmaceuticals: Optimizing Drug Discovery and Industry Operations: RSC Pharmaceutics.

[201] Smith DP, Oechsle O, Rawling MJ, Savory E, Lacoste AMB, Richardson PJ. Expert-Augmented Computational Drug Repurposing Identified Baricitinib as a Treatment for COVID-19. Front Pharmacol. 2021;Volume 12–2021. 10.3389/fphar.2021.709856 PMC8356560 34393789

[202] Richardson P, Griffin I, Tucker C, Smith D, Oechsle O, Phelan A, et al. Baricitinib as potential treatment for 2019-nCoV acute respiratory disease. Lancet. 2020;395(10223):e30–1. 32032529 10.1016/S0140-6736(20)30304-4 PMC7137985

[203] Niarakis A, Laubenbacher R, An G, Ilan Y, Fisher J, Flobak Å, et al. Immune digital twins for complex human pathologies: applications, limitations, and challenges. NPJ Syst Biol Appl. 2024;10(1):141. 39616158 10.1038/s41540-024-00450-5 PMC11608242

[204] Wang K, Parrott NJ, Lavé T. Embracing the future of medicine with virtual patients. Drug Discov Today. 2025. 10.1016/j.drudis.2025.104322. 40032136 10.1016/j.drudis.2025.104322

[205] Debnath R, Ikbal AMA, Choudhury A, Mandal SC, Palit P. Artificial Intelligence: A Major Landmark in the Novel Drug Discovery Pathway for the Remarkable Advancement in the Healthcare System. Concepts in Pharmaceutical Biotechnology and Drug Development. Singapore: Springer Nature Singapore. pp. 413–36.

[206] Lu MY, Chuang WL, Yu ML. The role of artificial intelligence in the management of liver diseases. Kaohsiung J Med Sci. 2024;40(11):962–71. 39440678 10.1002/kjm2.12901 PMC11895080

[207] Gangwal A, Lavecchia A. Artificial intelligence in preclinical research: enhancing digital twins and organ-on-chip to reduce animal testing. Drug Discov Today. 2025. 10.1016/j.drudis.2025.104360. 40252989 10.1016/j.drudis.2025.104360

[208] Sen S, Kilic KD. Digital Twins in Medicine, AI-Driven Personalized Healthcare, and Predictive Analytics. AI-Powered Digital Twins for Predictive Healthcare: Creating Virtual Replicas of Humans: IGI Global Scientific Publishing. pp. 359–96.

[209] Wang J, Tian Y, Zhou T, Tong D, Ma J, Li J. A survey of artificial intelligence in rheumatoid arthritis. Rheumatology and Immunology Research. 2023;4(2):69–77. 37485476 10.2478/rir-2023-0011 PMC10362600

[210] Ullah R, Sarwar N, Alatawi MN, Alsadhan AA, Salamah Alwageed H, Khan M, et al. Advancing personalized diagnosis and treatment using deep learning architecture. Front Med (Lausanne). 2025;12:1545528. 40212269 10.3389/fmed.2025.1545528 PMC11983605

[211] Rigi A, Harati K, Abbaspour M, Fattahpour SF, Hosseini P, Fard AM et al. AI and Deep Learning in Understanding the Etiology and Pathogenesis of Autoimmune Diseases. Kindle.4(1):1–182.

[212] Chen YM, Hsiao TH, Lin CH, Fann YC. Unlocking precision medicine: clinical applications of integrating health records, genetics, and immunology through artificial intelligence. J Biomed Sci. 2025;32(1):16. 39915780 10.1186/s12929-024-01110-w PMC11804102

[213] Basha SA, Hanirex DK, editors. Artificial Intelligence in Immunology: Predictive Models for Immune System Disorders. 2024 Global Conference on Communications and Information Technologies (GCCIT: IEEE.

[214] Fritzler MJ, Mahler M. Precision medicine as an approach to autoimmune diseases. Precision Medicine and Artificial Intelligence: Academic. pp. 39–63.

[215] Swetlik C, Bove R, McGinley M. Clinical and research applications of the electronic medical record in multiple sclerosis: a narrative review of current uses and future applications. Int J MS Care. 2022;24(6):287–94. 36545651 10.7224/1537-2073.2022-066 PMC9749832

[216] Houssein EH, Hosney ME, Emam MM, Younis EM, Ali AA, Mohamed WM. Soft computing techniques for biomedical data analysis: open issues and challenges. Artif Intell Rev. 2023;56(Suppl 2):2599–649.

[217] Gupta NS, Kumar P. Perspective of artificial intelligence in healthcare data management: a journey towards precision medicine. Comput Biol Med. 2023;162:107051. 37271113 10.1016/j.compbiomed.2023.107051

[218] Li TZ, Still JM, Xu K, Lee HH, Cai LY, Krishnan AR, et al. Longitudinal Multimodal Transformer Integrating Imaging and Latent Clinical Signatures From Routine EHRs for Pulmonary Nodule Classification. Med Image Comput Comput Assist Interv. 2023;14221:649–59. 38779102 10.1007/978-3-031-43895-0_61 PMC11110542

[219] Rane N, Choudhary S, Rane J. Explainable artificial intelligence (XAI) in healthcare: Interpretable models for clinical decision support.

[220] Yarman BS, Rathore SPS. The Future of AI in Disease Detection—A Look at Emerging Trends and Future Directions in the Use of AI for Disease Detection and Diagnosis. AI in Disease Detection: Advancements and Applications. pp. 265–88.

[221] Chaudhary G. Unveiling the black box: bringing algorithmic transparency to AI. Masaryk Univ J Law Technol. 2024;18(1):93–122.

[222] Natrayan L, Socrates S, Bharathi GB, Aluvala S, editors. A Framework for Automated Diagnosis and Management of Autoimmune Disorders with Neural Networks. 2024 International Conference on Advancements in Smart, Secure and Intelligent Computing (ASSIC: IEEE.

[223] ŞAhiN E, Arslan NN, Özdemir D. Unlocking the black box: an in-depth review on interpretability, explainability, and reliability in deep learning. Neural Comput Appl. 2025;37(2):859–965.

[224] Mo Q, Bolideei M, Rong S-J, Luo J-H, Yang C-L, Lu W-Y, et al. GSK2334470 attenuates high salt-exacerbated rheumatoid arthritis progression by restoring Th17/Treg homeostasis. iScience. 2024. 10.1016/j.isci.2024.109798. 38947509 10.1016/j.isci.2024.109798 PMC11214488

[225] Sun Y, Lin J, Chen W. Artificial intelligence in rheumatoid arthritis. Rheumatology & Autoimmunity. 2025. 10.1002/rai2.12171.

[226] Momtazmanesh S, Nowroozi A, Rezaei N. Artificial intelligence in rheumatoid arthritis: current status and future perspectives: a state-of-the-art review. Rheumatol Ther. 2022;9(5):1249–304. 35849321 10.1007/s40744-022-00475-4 PMC9510088

[227] Sharma SD, Bluett J. Towards personalized medicine in rheumatoid arthritis. Open Access Rheumatol Res Rev. 2024. 10.2147/OARRR.S372610. 10.2147/OARRR.S372610 PMC11110814 38779469

[228] Alsaber AR, Al-Herz A, Alawadhi B, Doush IA, Setiya P, Al-Sultan AT, et al. Machine learning-based remission prediction in rheumatoid arthritis patients treated with biologic disease-modifying anti-rheumatic drugs: findings from the Kuwait rheumatic disease registry. Front Big Data. 2024;7:1406365. 39421133 10.3389/fdata.2024.1406365 PMC11484091

[229] Koo BS, Eun S, Shin K, Yoon H, Hong C, Kim DH et al. Machine learning model for identifying important clinical features for predicting remission in patients with rheumatoid arthritis treated with biologics. Arthritis Res Therapy.23:1–10. 10.1186/s13075-021-02567-y PMC8259419 34229736

[230] Benavent D, Carmona L, Llorente JFG, Montoro M, Ramirez S, Otón T, et al. Empowering rheumatology through digital health technologies: contributions and barriers. Exploration of Musculoskeletal Diseases. 2024;2(2):92–105.

[231] Capobianco E. High-dimensional role of AI and machine learning in cancer research. Br J Cancer. 2022;126(4):523–32. 35013580 10.1038/s41416-021-01689-z PMC8854697

[232] Klareskog L, Rönnelid J, Saevarsdottir S, Padyukov L, Alfredsson L. The importance of differences: on environment and its interactions with genes and immunity in the causation of rheumatoid arthritis. J Intern Med. 2020;287(5):514–33. 32176395 10.1111/joim.13058

[233] Zhang B, Shi H, Wang H. Machine learning and AI in cancer prognosis, prediction, and treatment selection: a critical approach. J Multidiscip Healthc. 2023. 10.2147/JMDH.S410301. 37398894 10.2147/JMDH.S410301 PMC10312208

[234] Imtiaz M, Shah SAA, Rehman Z. A review of arthritis diagnosis techniques in the artificial intelligence era: Current trends and research challenges. Neurosci Inf.2(4):100079&#8211.

[235] Afrazeh F, Shomalzadeh M. Revolutionizing Arthritis Care with Artificial Intelligence: A Comprehensive Review of Diagnostic, Prognostic, and Treatment Innovations. Int J Appl Data Sci Eng Health.1(2):7–17.

[236] Mate GS, Kureshi AK, Singh BK. An efficient CNN for hand X-ray classification of rheumatoid arthritis. J Healthc Eng. 2021;1:6712785. 10.1155/2021/6712785 PMC8219419 34221300

[237] Bai L, Zhang Y, Wang P, Zhu X, Xiong JW, Cui L. Improved diagnosis of rheumatoid arthritis using an artificial neural network. Sci Rep. 2022;12(1):9810. 35697754 10.1038/s41598-022-13750-9 PMC9192742

[238] Subhash MG, Kureshi AK. An efficient CNN for hand X-ray classification of rheumatoid arthritis. Microprocess Microsyst.104822.

[239] Khan FN, Asim M, Qureshi MI. Artificial intelligence in the diagnosis and treatment of rheumatoid arthritis: Current status and prospects. Artificial Intelligence and Autoimmune Diseases: Applications in the Diagnosis, Prognosis, and Therapeutics. pp. 193–221.

[240] Plant D, Maciejewski M, Smith S, Nair N, Rheumatoid Arthritis Consortium MTU, Group RS, et al. Profiling of gene expression biomarkers as a classifier of methotrexate nonresponse in patients with rheumatoid arthritis. Arthritis Rheumatol. 2019;71(5):678–84. 30615300 10.1002/art.40810 PMC9328381

[241] Jian C, Chen S, Wang Z, Zhou Y, Zhang Y, Li Z, et al. Predicting delayed methotrexate elimination in pediatric acute lymphoblastic leukemia patients: an innovative web-based machine learning tool developed through a multicenter, retrospective analysis. BMC Med Inform Decis Mak. 2023;23(1):148. 37537590 10.1186/s12911-023-02248-7 PMC10398990

[242] Gosselt HR, Verhoeven MM, Bulatović-Ćalasan M, Welsing PM, Rotte MC, Hazes JM et al. Complex machine-learning algorithms and multivariable logistic regression on par in the prediction of insufficient clinical response to methotrexate in rheumatoid arthritis. J personalized Med.11(1):44&#8211. 10.3390/jpm11010044 PMC7828730 33466633

[243] Parsaei A, Moradi S, Masoumi M, Davatchi F, Najafi A, Kooshki AM, et al. Predictive value of erythrocyte sedimentation rate and C-reactive protein in Behcet’s disease activity and manifestations: a cross-sectional study. BMC Rheumatol. 2022;6(1):9. 35144674 10.1186/s41927-021-00241-z PMC8832718

[244] Spiliopoulou A, Colombo M, Plant D, Nair N, Cui J, Coenen MJ, et al. Association of response to TNF inhibitors in rheumatoid arthritis with quantitative trait loci for CD40 and CD39. Ann Rheum Dis. 2019;78(8):1055–61. 31036624 10.1136/annrheumdis-2018-214877 PMC6669378

[245] Mehran MJ, Mohammadzadeh S, Bolideei M, Barzigar R, Haider KH, Jadgal N, et al. Artificial intelligence in drug discovery: integrative advances from data to therapeutic innovation. Drug Dev Res. 2026;87(2):e70229. 41630488 10.1002/ddr.70229

[246] Kahlon MS, Islam MM, Vashishat A, Raikwar S. Revolutionizing rheumatoid arthritis care: AI-infused herbal treatments and the road ahead. Curr Pharm Biotechnol. 2025;26(3):316–8. 38757330 10.2174/0113892010305528240506114408

[247] Ivanisevic T, Sewduth RN. Multi-omics integration for the design of novel therapies and the identification of novel biomarkers. Proteomes. 2023;11(4):34. 37873876 10.3390/proteomes11040034 PMC10594525

[248] Ramchandani M, Goyal AK. Integrating omics data for personalized medicine in treating psoriasis. Med Chem Res. 2025;34(2):340–56.

[249] Shi Y, Zhou M, Chang C, Jiang P, Wei K, Zhao J, et al. Advancing precision rheumatology: applications of machine learning for rheumatoid arthritis management. Front Immunol. 2024;15:1409555. 38915408 10.3389/fimmu.2024.1409555 PMC11194317

[250] Madrid-García A, Merino-Barbancho B, Rodríguez-González A, Fernández-Gutiérrez B, Rodríguez-Rodríguez L, Menasalvas-Ruiz E. Understanding the role and adoption of artificial intelligence techniques in rheumatology research: an in-depth review of the literature. Seminars in Arthritis and Rheumatism. 61: WB Saunders. p. 152213&#8211. 10.1016/j.semarthrit.2023.152213 37315379

[251] Du M, Li S, Jiang J, Ma X, Liu L, Wang T, et al. Advances in the pathogenesis and treatment strategies for type 1 diabetes mellitus. Int Immunopharmacol. 2025;148:114185. 39893858 10.1016/j.intimp.2025.114185

[252] Azab ME, Amer H, Mohammwd W, ElSeddek M. Leveraging Artificial Intelligence in the Diagnosis and Management of Diabetic Foot Ulcers: A Review of Current Trends and Future Directions. Int J Telecommunications.5(01):1–26.

[253] Schwarz PE, Li J, Lindstrom J, Tuomilehto J. Tools for predicting the risk of type 2 diabetes in daily practice. Horm Metab Res. 2009;41(02):86–97. 19021089 10.1055/s-0028-1087203

[254] Ljubic B, Hai AA, Stanojevic M, Diaz W, Polimac D, Pavlovski M, et al. Predicting complications of diabetes mellitus using advanced machine learning algorithms. J Am Med Inform Assoc. 2020;27(9):1343–51. 32869093 10.1093/jamia/ocaa120 PMC7647294

[255] Dagliati A, Marini S, Sacchi L, Cogni G, Teliti M, Tibollo V, et al. Machine learning methods to predict diabetes complications. J Diabetes Sci Technol. 2018;12(2):295–302. 28494618 10.1177/1932296817706375 PMC5851210

[256] Behera A. Use of artificial intelligence for management and identification of complications in diabetes. Clin Diabetol.10(2):221–5.

[257] Kosaki K, Tarumi T, Sugawara J, Tanahashi K, Kumagai H, Matsui M, et al. Renal hemodynamics across the adult lifespan: relevance of flow pulsatility to chronic kidney disease. Exp Gerontol. 2021;152:111459. 34171394 10.1016/j.exger.2021.111459

[258] Khanam JJ, Foo SY. A comparison of machine learning algorithms for diabetes prediction. ICT Express. 2021;7(4):432–9.

[259] Rajendra P, Latifi S. Prediction of diabetes using logistic regression and ensemble techniques. Comput Methods Programs Biomed Update. 2021;1:100032.

[260] Ganie SM, Pramanik PKD, Bashir Malik M, Mallik S, Qin H. An ensemble learning approach for diabetes prediction using boosting techniques. Front Genet. 2023;14:1252159. 37953921 10.3389/fgene.2023.1252159 PMC10639159

[261] Cescon M, DeSalvo DJ, Ly TT, Maahs DM, Messer LH, Buckingham BA, et al. Early detection of infusion set failure during insulin pump therapy in type 1 diabetes. J Diabetes Sci Technol. 2016;10(6):1268–76. 27621142 10.1177/1932296816663962 PMC5094340

[262] Russell WE, Bundy BN, Anderson MS, Cooney LA, Gitelman SE, Goland RS, et al. Abatacept for Delay of Type 1 Diabetes Progression in Stage 1 Relatives at Risk: A Randomized, Double-Masked, Controlled Trial. Diabetes Care. 2023;46(5):1005–13. 36920087 10.2337/dc22-2200 PMC10154649

[263] Orban T, Bundy B, Becker DJ, DiMeglio LA, Gitelman SE, Goland R, et al. Co-stimulation modulation with abatacept in patients with recent-onset type 1 diabetes: a randomised, double-blind, placebo-controlled trial. Lancet. 2011;378(9789):412–9. 21719096 10.1016/S0140-6736(11)60886-6 PMC3462593

[264] Seelig E, Howlett J, Porter L, Truman L, Heywood J, Kennet J et al. The DILfrequency study is an adaptive trial designed to identify the optimal IL-2 dosing regimen in patients with type 1 diabetes. JCI insight.3(19):99306&#8211. 10.1172/jci.insight.99306 PMC6237447 30282826

[265] Waibel M, Wentworth JM, So M, Couper JJ, Cameron FJ, MacIsaac RJ, et al. Baricitinib and β-cell function in patients with new-onset type 1 diabetes. N Engl J Med. 2023;389(23):2140–50. 38055252 10.1056/NEJMoa2306691

[266] Rosenzwajg M, Salet R, Lorenzon R, Tchitchek N, Roux A, Bernard C, et al. Low-dose IL-2 in children with recently diagnosed type 1 diabetes: a phase I/II randomised, double-blind, placebo-controlled, dose-finding study. Diabetologia. 2020;63:1808–21. 32607749 10.1007/s00125-020-05200-w

[267] Tanoli Z, Vähä-Koskela M, Aittokallio T. Artificial intelligence, machine learning, and drug repurposing in cancer. Expert Opin Drug Discov. 2021;16(9):977–89. 33543671 10.1080/17460441.2021.1883585

[268] Hannelius U, Beam CA, Ludvigsson J. Efficacy of GAD-alum immunotherapy associated with HLA-DR3-DQ2 in recently diagnosed type 1 diabetes. Diabetologia. 2020;63:2177–81. 32754804 10.1007/s00125-020-05227-z PMC7476912

[269] Jacobsen LM, Newby BN, Perry DJ, Posgai AL, Haller MJ, Brusko TM. Immune mechanisms and pathways targeted in type 1 diabetes. Curr Diabetes Rep. 2018;18:1–20. 10.1007/s11892-018-1066-5 PMC8053389 30168021

[270] Nowak C, Lind M, Sumnik Z, Pelikanova T, Nattero-Chavez L, Lundberg E et al. Intralymphatic GAD-alum (Diamyd^®^) improves glycemic control in type 1 diabetes with HLA DR3-DQ2. J Clin Endocrinol Metabolism.107(9):2644–51. 10.1210/clinem/dgac343 PMC9721339 35665810

[271] Freda PJ, Ghosh A, Zhang E, Luo T, Chitre AS, Polesskaya O et al. Automated quantitative trait locus analysis (AutoQTL. BioData Min.16(1):14&#8211. 10.1186/s13040-023-00331-3 PMC10088184 37038201

[272] Sanchez-Trincado JL, Gomez-Perosanz M, Reche PA. Fundamentals and methods for T- and B-cell epitope prediction. J Immunol Res.2017(1):2680160&#8211. 10.1155/2017/2680160 PMC5763123 29445754

[273] Kacen A, Javitt A, Kramer MP, Morgenstern D, Tsaban T, Shmueli MD, et al. Post-translational modifications reshape the antigenic landscape of the MHC I immunopeptidome in tumors. Nat Biotechnol. 2023;41(2):239–51. 36203013 10.1038/s41587-022-01464-2 PMC11197725

[274] Katayama Y, Yokota R, Akiyama T, Kobayashi TJ. Machine learning approaches to TCR repertoire analysis. Front Immunol. 2022;13:858057. 35911778 10.3389/fimmu.2022.858057 PMC9334875

[275] Herrath M, Bain SC, Bode B, Clausen JO, Coppieters K, Gaysina L et al. Anti-interleukin-21 antibody and liraglutide for the preservation of β-cell function in adults with recent-onset type 1 diabetes: a randomised, double-blind, placebo-controlled, phase 2 trial. Lancet Diabetes Endocrinol.9(4):212–24. 10.1016/S2213-8587(21)00019-X 33662334

[276] Chou WC, Lin Z. Machine learning and artificial intelligence in physiologically based pharmacokinetic modeling. Toxicol Sci. 2023;191(1):1–14. 36156156 10.1093/toxsci/kfac101 PMC9887681

[277] Long SA, Rieck M, Sanda S, Bollyky JB, Samuels PL, Goland R, et al. Rapamycin/IL-2 combination therapy in patients with type 1 diabetes augments Tregs yet transiently impairs β-cell function. Diabetes. 2012;61(9):2340–8. 22721971 10.2337/db12-0049 PMC3425404

[278] Karakose E, Wang X, Wang P, Carcamo S, Demircioglu D, Lambertini L et al. Single Cell RNA-Seq Analysis of Regenerative Drug-Treated Human Pancreatic Islets Identifies A Cycling Alpha Cell Population As Key Beta Cell Progenitors. 10.1016/j.xcrm.2024.101832 PMC11722108 39626675

[279] Furukawa A, Wisel SA, Tang Q. Impact of immune-modulatory drugs on regulatory T cells. Transplantation.100(11):2288–300. 10.1097/TP.0000000000001379 PMC5077666 27490409

[280] Tsonkova VG, Sand FW, Wolf XA, Grunnet LG, Ringgaard AK, Ingvorsen C, et al. The EndoC-βH1 cell line is a valid model of human beta cells and applicable for screenings to identify novel drug target candidates. Mol Metab. 2018;8:144–57. 29307512 10.1016/j.molmet.2017.12.007 PMC5985049

[281] Deligne C, You S, Mallone R. Personalized immunotherapies for type 1 diabetes: who, what, when, and how? J Pers Med. 2022;12(4):542. 35455658 10.3390/jpm12040542 PMC9031881

[282] Pescovitz MD, Greenbaum CJ, Krause-Steinrauf H, Becker DJ, Gitelman SE, Goland R, et al. Rituximab, B-lymphocyte depletion, and preservation of beta-cell function. N Engl J Med. 2009;361(22):2143–52. 19940299 10.1056/NEJMoa0904452 PMC6410357

[283] Noble JA. Immunogenetics of type 1 diabetes: a comprehensive review. J Autoimmun. 2015;64:101–12. 26272854 10.1016/j.jaut.2015.07.014

[284] Shapiro MR, Tallon EM, Brown ME, Posgai AL, Clements MA, Brusko TM. Leveraging artificial intelligence and machine learning to accelerate the discovery of disease-modifying therapies in type 1 diabetes. Diabetologia.1–18. 10.1007/s00125-024-06339-6 PMC11832708 39694914

[285] Cappon G, Facchinetti A. Digital twins in type 1 diabetes: a systematic review. J Diabetes Sci Technol. 2024. 10.1177/19322968241262112. 38887022 10.1177/19322968241262112 PMC11572256

[286] Rajpurkar P, Chen E, Banerjee O, Topol EJ. AI in health and medicine. Nat Med. 2022;28(1):31–8. 35058619 10.1038/s41591-021-01614-0

[287] Karim MR, Islam T, Shajalal M, Beyan O, Lange C, Cochez M, et al. Explainable AI for bioinformatics: methods, tools, and applications. Brief Bioinform. 2023;24(5):236. 10.1093/bib/bbad236 37478371

[288] Kırboğa KK, Abbasi S, Küçüksille EU. Explainability and white box in drug discovery. Chem Biol Drug Des. 2023;102(1):217–33. 37105727 10.1111/cbdd.14262

[289] Vasquez VM Jr., McCabe M, McKee JC, Siby S, Hussain U, Faizuddin F, et al. Transforming cancer care: a narrative review on leveraging artificial intelligence to advance immunotherapy in underserved communities. J Clin Med. 2025. 10.3390/jcm14155346. 10.3390/jcm14155346 PMC12347673 40806968

[290] Arora A, Alderman JE, Palmer J, Ganapathi S, Laws E, McCradden MD, et al. The value of standards for health datasets in artificial intelligence-based applications. Nat Med. 2023;29(11):2929–38. 37884627 10.1038/s41591-023-02608-w PMC10667100

[291] Chen RJ, Wang JJ, Williamson DFK, Chen TY, Lipkova J, Lu MY, et al. Algorithmic fairness in artificial intelligence for medicine and healthcare. Nat Biomed Eng. 2023;7(6):719–42. 37380750 10.1038/s41551-023-01056-8 PMC10632090

[292] Fahim YA, Hasani IW, Kabba S, Ragab WM. Artificial intelligence in healthcare and medicine: clinical applications, therapeutic advances, and future perspectives. Eur J Med Res. 2025;30(1):848. 40988064 10.1186/s40001-025-03196-w PMC12455834

[293] Albani FG, Alghamdi SS, Almutairi MM, Alqahtani T. Artificial intelligence-driven innovations in oncology drug discovery: transforming traditional pipelines and enhancing drug design. Drug Des Devel Ther. 2025;19:5685–707. 40626099 10.2147/DDDT.S509769 PMC12232943

[294] Maiorano MFP, Cormio G, Loizzi V, Maiorano BA. Artificial intelligence in ovarian cancer: a systematic review and meta-analysis of predictive AI models in genomics, radiomics, and immunotherapy. AI. 2025;6(4):84.

[295] Fraser AG, Biasin E, Bijnens B, Bruining N, Caiani EG, Cobbaert K, et al. Artificial intelligence in medical device software and high-risk medical devices - a review of definitions, expert recommendations and regulatory initiatives. Expert Rev Med Devices. 2023;20(6):467–91. 37157833 10.1080/17434440.2023.2184685

[296] Kelly CJ, Karthikesalingam A, Suleyman M, Corrado G, King D. Key challenges for delivering clinical impact with artificial intelligence. BMC Med. 2019;17(1):195. 31665002 10.1186/s12916-019-1426-2 PMC6821018

[297] Abbas SR, Abbas Z, Zahir A, Lee SW. Federated learning in smart healthcare: a comprehensive review on privacy, security, and predictive analytics with IoT integration. Healthcare. 2024;12(24):2587. 39766014 10.3390/healthcare12242587 PMC11728217

[298] Amezquita RA, Lun ATL, Becht E, Carey VJ, Carpp LN, Geistlinger L, et al. Orchestrating single-cell analysis with Bioconductor. Nat Methods. 2020;17(2):137–45. 31792435 10.1038/s41592-019-0654-x PMC7358058

[299] Rieke N, Hancox J, Li W, Milletarì F, Roth HR, Albarqouni S, et al. The future of digital health with federated learning. NPJ Digit Med. 2020;3(1):119. 33015372 10.1038/s41746-020-00323-1 PMC7490367

[300] Shen S, Qi W, Liu X, Zeng J, Li S, Zhu X, et al. From virtual to reality: innovative practices of digital twins in tumor therapy. J Transl Med. 2025;23(1):348. 40108714 10.1186/s12967-025-06371-z PMC11921680

[301] Chaudhuri A, Pash G, Hormuth DA, Lorenzo G, Kapteyn M, Wu C et al. Predictive digital twin for optimizing patient-specific radiotherapy regimens under uncertainty in high-grade gliomas. Front Artif Intell. 2023;Volume 6–2023. 10.3389/frai.2023.1222612 PMC10598726 37886348

[302] Samei E. The future of in silico trials and digital twins in medicine. PNAS Nexus. 2025;4(5):pgaf123. 40313535 10.1093/pnasnexus/pgaf123 PMC12043051

[303] Liu X, Cruz Rivera S, Moher D, Calvert MJ, Denniston AK. Reporting guidelines for clinical trial reports for interventions involving artificial intelligence: the CONSORT-AI extension. Nat Med. 2020;26(9):1364–74. 32908283 10.1038/s41591-020-1034-x PMC7598943

[304] Zhang C-c, Feng H-r, Zhu J, Hong W-f. Application of spatial and single-cell omics in tumor immunotherapy biomarkers. Labmed Discov. 2025. 10.1016/j.lmd.2025.100076.

[305] Li Y, Wu X, Fang D, Luo Y. Informing immunotherapy with multi-omics driven machine learning. NPJ Digit Med. 2024;7(1):67. 38486092 10.1038/s41746-024-01043-6 PMC10940614

[306] Wei F, Azuma K, Nakahara Y, Saito H, Matsuo N, Tagami T, et al. Machine learning for prediction of immunotherapeutic outcome in non-small-cell lung cancer based on circulating cytokine signatures. J Immunother Cancer. 2023. 10.1136/jitc-2023-006788. 37433717 10.1136/jitc-2023-006788 PMC10347453

[307] Zhao Z, Li Y, Wu Y, Chen R. Deep learning-based model for predicting progression in patients with head and neck squamous cell carcinoma. Cancer Biomark. 2020;27(1):19–28. 31658045 10.3233/CBM-190380 PMC12662277

[308] He Q, Xiao B, Tan Y, Wang J, Tan H, Peng C, et al. Integrated multicenter deep learning system for prognostic prediction in bladder cancer. npj Precision Oncol. 2024;8(1):233. 10.1038/s41698-024-00731-6 PMC11484793 39414931

[309] Nie W, Jiang Y, Yao L, Zhu X, Al-Danakh AY, Liu W, et al. Prediction of bladder cancer prognosis and immune microenvironment assessment using machine learning and deep learning models. Heliyon. 2024;10(23):e39327. 39687145 10.1016/j.heliyon.2024.e39327 PMC11647853

[310] Sammut S-J, Crispin-Ortuzar M, Chin S-F, Provenzano E, Bardwell HA, Ma W, et al. Multi-omic machine learning predictor of breast cancer therapy response. Nature. 2022;601(7894):623–9. 34875674 10.1038/s41586-021-04278-5 PMC8791834

[311] Wang HN, An JH, Wang FQ, Hu WQ, Zong L. Predicting gastric cancer survival using machine learning: a systematic review. World J Gastrointest Oncol. 2025;17(5):103804. 40487963 10.4251/wjgo.v17.i5.103804 PMC12142261

[312] Zhou C-M, Wang Y, Yang J-J, Zhu Y. Predicting postoperative gastric cancer prognosis based on inflammatory factors and machine learning technology. BMC Med Inform Decis Mak. 2023;23(1):53. 37004065 10.1186/s12911-023-02150-2 PMC10067164

[313] Liu L, Zhang Y, Zhao X-h, Zhu M, Liang J-w, Jiang Z-y, et al. Predicting clinical prognosis in gastric cancer using deep learning-based analysis of tissue pathomics images. Comput Methods Programs Biomed. 2025;269:108895. 40513510 10.1016/j.cmpb.2025.108895

[314] Liu X, Tao P, Su H, Li Y. Machine learning-random forest model was used to construct gene signature associated with cuproptosis to predict the prognosis of gastric cancer. Sci Rep. 2025;15(1):4170. 39905263 10.1038/s41598-025-88812-9 PMC11794614

[315] Li HJ, Qiu ZB, Wang MM, Zhang C, Hong HZ, Fu R, et al. Radiomics-based support vector machine distinguishes molecular events driving the progression of lung adenocarcinoma. J Thorac Oncol. 2025;20(1):52–64. 39306192 10.1016/j.jtho.2024.09.1431

[316] Mazzaschi G, Milanese G, Pagano P, Madeddu D, Gnetti L, Trentini F, et al. Integrated CT imaging and tissue immune features disclose a radio-immune signature with high prognostic impact on surgically resected NSCLC. Lung Cancer. 2020;144:30–9. 32361033 10.1016/j.lungcan.2020.04.006

[317] Tong H, Sun J, Fang J, Zhang M, Liu H, Xia R et al. A Machine Learning Model Based on PET/CT Radiomics and Clinical Characteristics Predicts Tumor Immune Profiles in Non-Small Cell Lung Cancer: A Retrospective Multicohort Study. Front Immunol. 2022;Volume 13–2022. 10.3389/fimmu.2022.859323 PMC9105942 35572597

[318] Li C, Zhou Z, Hou L, Hu K, Wu Z, Xie Y, et al. A novel machine learning model for efficacy prediction of immunotherapy-chemotherapy in NSCLC based on CT radiomics. Comput Biol Med. 2024;178:108638. 38897152 10.1016/j.compbiomed.2024.108638

[319] Chuang C-C, Liu Y-C, Ou Y-Y. DeepNeoAG: Neoantigen epitope prediction from melanoma antigens using a synergistic deep learning model combining protein language models and multi-window scanning convolutional neural networks. Int J Biol Macromol. 2024;281:136252. 39366619 10.1016/j.ijbiomac.2024.136252

[320] Tripathi S, Moyer EJ, Augustin AI, Zavalny A, Dheer S, Sukumaran R, et al. RadGenNets: Deep learning-based radiogenomics model for gene mutation prediction in lung cancer. Inf Med Unlock. 2022;33:101062.

[321] Machaca V, Goyzueta V, Cruz MG, Sejje E, Pilco LM, López J, et al. Transformers meets neoantigen detection: a systematic literature review. J Integr Bioinform. 2024. 10.1515/jib-2023-0043. 38960869 10.1515/jib-2023-0043 PMC11377031

[322] Menyhárt O, Győrffy B. Multi-omics approaches in cancer research with applications in tumor subtyping, prognosis, and diagnosis. Comput Struct Biotechnol J. 2021;19:949–60. 33613862 10.1016/j.csbj.2021.01.009 PMC7868685

[323] Ju H, Kim K, Kim BI, Woo S-K. Graph neural network model for prediction of non-small cell lung cancer lymph node metastasis using protein–protein interaction network and 18F-FDG PET/CT radiomics. Int J Mol Sci. 2024;25(2):698. 38255770 10.3390/ijms25020698 PMC10815846

[324] Khan A, Sajid MZ, Khan NA, Youssef A, Abbas Q. CAD-Skin: a hybrid convolutional neural network–autoencoder framework for precise detection and classification of skin lesions and cancer. Bioengineering. 2025;12(4):326. 40281686 10.3390/bioengineering12040326 PMC12025204

[325] Fang X, Chong CF, Wong KL, Simões M, Ng BK. Investigating the key principles in two-step heterogeneous transfer learning for early laryngeal cancer identification. Sci Rep. 2025;15(1):2146. 39820368 10.1038/s41598-024-84836-9 PMC11739633

[326] Yang R, Li W, Yu S, Wu Z, Zhang H, Liu X, et al. Enhanced NSCLC subtyping and staging through attention-augmented multi-task deep learning: a novel diagnostic tool. Int J Med Inform. 2025;193:105694. 39515045 10.1016/j.ijmedinf.2024.105694

[327] Wagner J, Oldenburg J, Nath N, Simm S. Explainable AI model reveals informative mutational signatures for cancer-type classification. Cancers. 2025;17(11):1731. 40507213 10.3390/cancers17111731 PMC12153866

[328] Abas Mohamed Y, Ee Khoo B, Shahrimie Mohd Asaari M, Ezane Aziz M, Rahiman Ghazali F. Decoding the black box: explainable AI (XAI) for cancer diagnosis, prognosis, and treatment planning-a state-of-the art systematic review. Int J Med Inform. 2025;193:105689. 39522406 10.1016/j.ijmedinf.2024.105689

[329] Singhal A, Agrawal KK, Quezada A, Aguiñaga AR, Jiménez S, Yadav SP. Explainable artificial intelligence (XAI) model for cancer image classification. CMES Comput Model Eng Sci. 2024;141(1):401–41.

[330] Maithresh A, Nikhil V, Saipuneeth H, Reddy GVS, editors. Exploring the Superiority of CapsNet over CNN For Early Detection of Lung Cancer A Comparative Analysis. 2023 International Conference on Inventive Computation Technologies (ICICT); 2023 26–28 April 2023.

[331] Bushara AR, Vinod Kumar RS, Kumar SS. An ensemble method for the detection and classification of lung cancer using computed tomography images utilizing a capsule network with Visual Geometry Group. Biomed Signal Process Control. 2023;85:104930.

[332] Ali M, Ali R. Multi-input dual-stream capsule network for improved lung and colon cancer classification. Diagnostics. 2021. 10.3390/diagnostics11081485. 34441419 10.3390/diagnostics11081485 PMC8393706

[333] Ochoa-Ornelas R, Gudiño-Ochoa A, García-Rodríguez JA. A hybrid deep learning and machine learning approach with Mobile-EfficientNet and Grey Wolf Optimizer for lung and colon cancer histopathology classification. Cancers. 2024;16(22):3791. 39594746 10.3390/cancers16223791 PMC11593226

[334] Gowthamy J, Ramesh S. A novel hybrid model for lung and colon cancer detection using pre-trained deep learning and KELM. Expert Syst Appl. 2024;252:124114.

[335] Debellotte O, Dookie RL, Rinkoo F, Kar A, Salazar González JF, Saraf P, et al. Artificial intelligence and early detection of breast, lung, and colon cancer: a narrative review. Cureus. 2025;17(2):e79199. 40125138 10.7759/cureus.79199 PMC11926462

[336] Krishnapriya S, Birudaraju H, Madhulatha M, Nagajyothi S, Ranadheer Kumar KS. Hybrid deep learning model for identifying the cancer type. BenchCouncil Trans Benchmarks Stand Eval. 2025;5(2):100211.

[337] Yang Y, Wang J, Xie F, Liu J, Shu C, Wang Y, et al. A convolutional neural network trained with dermoscopic images of psoriasis performed on par with 230 dermatologists. Comput Biol Med. 2021;139:104924. 34688173 10.1016/j.compbiomed.2021.104924

[338] Alhamdani MA, Hashim AN, editors. Classification of psoriasis and eczema based on Convolutional Neural Network. 2022 Iraqi International Conference on Communication and Information Technologies (IICCIT); 2022 7–8 Sept. 2022.

[339] Adebiyi MO, Destiny O, Olaniyan D, Olaniyan J, Oluwatomi D, editors. Systemic Lupus Erythematosus (SLE) Detection Using Deep Learning. 2024 International Conference on Science, Engineering and Business for Driving Sustainable Development Goals (SEB4SDG); 2024 2–4 April 2024.

[340] Rajimehr R, Farsiu S, Kouhsari LM, Bidari A, Lucas C, Yousefian S, et al. Prediction of lupus nephritis in patients with systemic lupus erythematosus using artificial neural networks. Lupus. 2002;11(8):485–92. 12220102 10.1191/0961203302lu226oa

[341] Bai L, Zhang Y, Wang P, Zhu X, Xiong J-W, Cui L. Improved diagnosis of rheumatoid arthritis using an artificial neural network. Sci Rep. 2022;12(1):9810. 35697754 10.1038/s41598-022-13750-9 PMC9192742

[342] Sharifmousavi SS, Borhani MS. Support vectors machine-based model for diagnosis of multiple sclerosis using the plasma levels of selenium, vitamin B12, and vitamin D3. Inf Med Unlocked. 2020;20:100382.

[343] Alivernini S, Cañete JD, Bacardit J, Kurowska-Stolarska M. Using explainable artificial intelligence to predict and forestall flare in rheumatoid arthritis. Nat Med. 2024;30(4):925–6. 38361121 10.1038/s41591-024-02818-w

[344] Pontiveros MJ, Solano GA, Tee CA, Tee ML, editors. Explainable machine learning applied to single-nucleotide polymorphisms for systemic lupus erythematosus prediction. 2020 11th International Conference on Information, Intelligence, Systems and Applications (IISA; 2020: IEEE.

[345] Lee C, Joo G, Shin S, Im H, Moon KW. Prediction of osteoporosis in patients with rheumatoid arthritis using machine learning. Sci Rep. 2023;13(1):21800. 38066096 10.1038/s41598-023-48842-7 PMC10709305

[346] Lee S, Kang S, Eun Y, Won H-H, Kim H, Lee J, et al. Machine learning-based prediction model for responses of bDMARDs in patients with rheumatoid arthritis and ankylosing spondylitis. Arthritis Res therapy. 2021;23:1–12. 10.1186/s13075-021-02635-3 PMC8501710 34627335

[347] Aijaz SF, Khan SJ, Azim F, Shakeel CS, Hassan U. Deep learning application for effective classification of different types of psoriasis. J Healthc Eng. 2022;2022(1):7541583. 35075392 10.1155/2022/7541583 PMC8783723

[348] de Ponce Leon-Sanchez ER, Dominguez-Ramirez OA, Herrera-Navarro AM, Rodriguez-Resendiz J, Paredes-Orta C, Mendiola-Santibañez JD. A deep learning approach for predicting multiple sclerosis. Micromachines. 2023;14(4):749. 37420982 10.3390/mi14040749 PMC10141207

[349] Williams DD, Ferro D, Mullaney C, Skrabonja L, Barnes MS, Patton SR, et al. An “all-data-on-hand” deep learning model to predict hospitalization for diabetic ketoacidosis in youth with type 1 diabetes: development and validation study. JMIR Diabetes. 2023;8:e47592. 37224506 10.2196/47592 PMC10394604

[350] Norgeot B, Glicksberg BS, Trupin L, Lituiev D, Gianfrancesco M, Oskotsky B, et al. Assessment of a deep learning model based on electronic health record data to forecast clinical outcomes in patients with rheumatoid arthritis. JAMA Netw Open. 2019;2(3):e190606-e. 30874779 10.1001/jamanetworkopen.2019.0606 PMC6484652

[351] Shehzadi N, Rehman A, Naz S, Rehman S, Khalifa F. Machine learning and deep learning based psoriasis recognition system: evaluation, management, prognosis—where we are and the way to the future. Artif Intell Rev. 2025;58(9):1–47.

[352] Pinto MF, Oliveira H, Batista S, Cruz L, Pinto M, Correia I, et al. Prediction of disease progression and outcomes in multiple sclerosis with machine learning. Sci Rep. 2020;10(1):21038. 33273676 10.1038/s41598-020-78212-6 PMC7713436

[353] Gu X-x, Jin Y, Fu T, Zhang X-m, Li T, Yang Y, et al. Relevant characteristics analysis using natural language processing and machine learning based on phenotypes and T-cell subsets in Systemic Lupus Erythematosus patients with anxiety. Front Psychiatry. 2021;12:793505. 34955935 10.3389/fpsyt.2021.793505 PMC8703039

[354] Deng Y, Pacheco JA, Chung A, Mao C, Smith JC, Zhao J et al. Natural language processing to identify lupus nephritis phenotype in electronic health records. arXiv preprint arXiv:211210821. 2021. 10.1186/s12911-024-02420-7 PMC10910523 38433189

[355] Omar M, Naffaa ME, Glicksberg BS, Reuveni H, Nadkarni GN, Klang E. Advancing rheumatology with natural language processing: insights and prospects from a systematic review. Rheumatol Adv Pract. 2024;8(4):rkae120. 39399162 10.1093/rap/rkae120 PMC11467191

[356] Gharehali S, Rahatabad FN, Einalou Z. Modeling multiple sclerosis at different levels using reinforcement learning. Int Clin Neurosci J. 2018;5(3):98–102.

[357] Shiezadeh Z, Sajedi H, Aflakie E. Diagnosis of rheumatoid arthritis using an ensemble learning approach. Comput Sci Inf Technol(CS IT). 2015;5(15):139–48.

[358] Wang L, Zhu L, Jiang J, Wang L, Ni W. Decision tree analysis for evaluating disease activity in patients with rheumatoid arthritis. J Int Med Res. 2021;49(10):03000605211053232. 34670422 10.1177/03000605211053232 PMC8543724

[359] Katsarou DN, Georga EI, Christou MA, Christou PA, Tigas S, Papaloukas C, et al. Optimizing hypoglycaemia prediction in type 1 diabetes with Ensemble Machine Learning modeling. BMC Med Inform Decis Mak. 2025;25(1):46. 39891137 10.1186/s12911-025-02867-2 PMC11783934

[360] Fukae J, Amasaki Y, Fujieda Y, Sone Y, Katagishi K, Horie T, et al. Pre-trained convolutional neural network with transfer learning by artificial illustrated images classify power Doppler ultrasound images of rheumatoid arthritis joints. J Int Med Res. 2025;53(2):03000605251318195. 39904596 10.1177/03000605251318195 PMC11795604

[361] Voigt I, Inojosa H, Dillenseger A, Haase R, Akgün K, Ziemssen T. Digital twins for multiple sclerosis. Front Immunol. 2021;12:669811. 34012452 10.3389/fimmu.2021.669811 PMC8128142

[362] Cen S, Gebregziabher M, Moazami S, Azevedo CJ, Pelletier D. Toward precision medicine using a “digital twin” approach: modeling the onset of disease-specific brain atrophy in individuals with multiple sclerosis. Sci Rep. 2023;13(1):16279. 37770560 10.1038/s41598-023-43618-5 PMC10539386

[363] Cappon G, Vettoretti M, Sparacino G, Del Favero S, Facchinetti A. Replaybg: a digital twin-based methodology to identify a personalized model from type 1 diabetes data and simulate glucose concentrations to assess alternative therapies. IEEE Trans Biomed Eng. 2023;70(11):3227–38. 37368794 10.1109/TBME.2023.3286856

